# De Novo Generation and Identification of Novel Compounds with Drug Efficacy Based on Machine Learning

**DOI:** 10.1002/advs.202307245

**Published:** 2024-01-10

**Authors:** Dakuo He, Qing Liu, Yan Mi, Qingqi Meng, Libin Xu, Chunyu Hou, Jinpeng Wang, Ning Li, Yang Liu, Huifang Chai, Yanqiu Yang, Jingyu Liu, Lihui Wang, Yue Hou

**Affiliations:** ^1^ College of Information Science and Engineering State Key Laboratory of Synthetical Automation for Process Industries Northeastern University Shenyang 110819 China; ^2^ Key Laboratory of Bioresource Research and Development of Liaoning Province College of Life and Health Sciences National Frontiers Science Center for Industrial Intelligence and Systems Optimization Northeastern University Shenyang 110169 China; ^3^ Key Laboratory of Data Analytics and Optimization for Smart Industry Ministry of Education Northeastern University Shenyang 110169 China; ^4^ School of Traditional Chinese Materia Medica Key Laboratory for TCM Material Basis Study and Innovative Drug Development of Shenyang City Shenyang Pharmaceutical University Shenyang 110016 China; ^5^ Key Laboratory of Structure‐Based Drug Design & Discovery of Ministry of Education Shenyang Pharmaceutical University Shenyang 110016 China; ^6^ School of Pharmacy Guizhou University of Traditional Chinese Medicine Guiyang 550025 China; ^7^ Department of Pharmacology Shenyang Pharmaceutical University Shenyang 110016 China

**Keywords:** drug efficacy, machine learning, de novo design, lead compound

## Abstract

One of the main challenges in small molecule drug discovery is finding novel chemical compounds with desirable activity. Traditional drug development typically begins with target selection, but the correlation between targets and disease remains to be further investigated, and drugs designed based on targets may not always have the desired drug efficacy. The emergence of machine learning provides a powerful tool to overcome the challenge. Herein, a machine learning‐based strategy is developed for de novo generation of novel compounds with drug efficacy termed DTLS (Deep Transfer Learning‐based Strategy) by using dataset of disease‐direct‐related activity as input. DTLS is applied in two kinds of disease: colorectal cancer (CRC) and Alzheimer's disease (AD). In each case, novel compound is discovered and identified in in vitro and in vivo disease models. Their mechanism of actionis further explored. The experimental results reveal that DTLS can not only realize the generation and identification of novel compounds with drug efficacy but also has the advantage of identifying compounds by focusing on protein targets to facilitate the mechanism study. This work highlights the significant impact of machine learning on the design of novel compounds with drug efficacy, which provides a powerful new approach to drug discovery.

## Graphical abstract

A machine learning‐based strategy for de novo generation of novel compounds with drug efficacy termed DTLS is proposed, which exhibits the advantage of directly generating molecules with desired drug efficacy by taking dataset of disease‐direct‐related activity, independent of target protein, as input, and proven to be effective for rapid generation and identification of desired lead compounds evidenced by wet experiments.

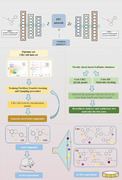

## 1 Introduction

The drug development process is extremely complex, lengthy, and expensive.^[^ ^1^, ^2^ ^]^ Advanced machine learning techniques have great potential to speed up this process and reduce the financial cost by rapidly identifying molecules with drug efficacy.^[^ ^3^, ^4^, ^5^, ^6^, ^7^ ^]^ Currently, most research has mainly focused on the design and application of machine learning algorithms and proof‐of‐concept studies, few of the novel molecules designed by generative models have been synthesized and investigated via in vitro and in vivo experiments.^[^ ^8^, ^9^, ^10^, ^11^, ^12^ ^]^

The goal of drug development is to design novel compounds with drug efficacy, which is one of the main challenges in small molecule drug discovery. Several recent research studies have used deep learning methods to establish drug efficacy prediction models to screen existing compound databases. For example, Stokes et al. proposed an antibiotic prediction model based on deep learning, which was used to predict the antibiotic activity of molecules in Drug Repurposing Hub and used to discover a known molecule for treating *Escherichia coli* infections.^[^ ^13^ ^]^ The Xie group proposed a deep learning model for drug efficacy prediction from a natural product library and FDA‐approved library.^[^ ^14^ ^]^ These studies, based on deep learning methods, focused on the screen of existing compounds, and no novel compounds with drug efficacy were designed.

In the present study, we propose a machine learning‐based‐strategy termed the Deep Transfer Learning‐based Strategy (DTLS) to generate novel compounds with drug efficacy. Our DTLS includes five stages. 1) A variational autoencoder coupled with a feature property correlation (VAE_FPC) network was trained as a molecule generation model to generate chemically valid and drug‐like molecules using the reprocessed ChEMBL database. 2) Quantitative or qualitative activity prediction models based on machine learning and dataset of disease‐direct‐related activity were constructed. 3) Partition recurrent transfer learning (PRTL) was trained on the VAE_FPC model using a disease‐direct‐related activity dataset to generate novel molecules with desirable properties. 4) Novel molecules with potential drug efficacy were screened using a drug efficacy‐based screening or target‐based screening strategy. 5) Novel molecules with potential drug efficacy were synthesized, followed by in vitro and in vivo studies and to further explore the compound‐involved mechanism of action. The developed procedure was performed for colorectal cancer (CRC) and Alzheimer's disease (AD), and has enabled the successful discovery of novel structured lead compounds for both diseases.

## 2 Results

### 2.1 The Architecture of De Novo Design of Novel Structured Lead Compounds Based on Machine Learning

**Figure 1:**
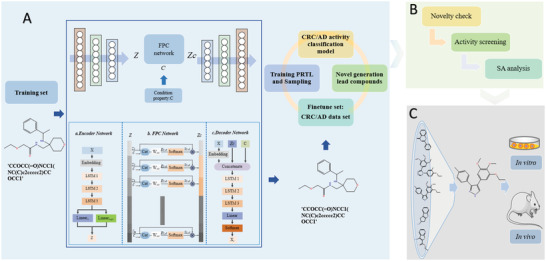
The architecture of de novo design of novel structured lead compounds based on machine learning. A) DTLS contain VAE_FPC and PRTL network. VAE_FPC network was trained using a preprocessed dataset to generate chemically valid and drug‐like molecules. PRTL was proposed to generate novel structured lead compounds for specific targets. B) Novelty screening was performed using the SciFinder database. The SA score was used to evaluate the synthetic feasibility of the molecules. We selected the molecules with the lowest SA score from Top 10 and Top 11–20, respectively. Retrosynthetic analysis and route of synthesis were performed. C) An in vitro cell model was used to determine the empirical IC_50_ values of the novel compounds and several known compounds were tested for comparison. Based on the characteristics of the specific disease, an in vivo animal model was established to confirm the efficacy of the lead compounds.

Module 1: A molecule generation model was trained based on the VAE_FPC network to generate chemically valid and drug‐like molecules, in which the input was a simplified molecular input line entry system (SMILES). The SMILES of the small molecule were then converted to latent vectors with continuous fixed dimensions using the Encoder network. The FPC network learns the correlation between the condition property and latent vectors. The decoder network was used to convert the latent vectors with the condition property into the molecule's SMILES (**Figure** **1**A).

Module 2: An activity classification model was constructed to classify the molecule as active or inactive against a specific disease, which involved two key factors: selection of the representations of the molecule and modeling methods. Three different molecule descriptors (Avalon, ECFP, Rdkit descriptor) were applied as molecule representations coupled with the three most widely used ML approaches (random forests, support vector machine, and gradient boosting decision tree) to construct an activity classification model against a specific disease, and the optimal model was selected for the subsequent molecule screening process using comparative results (Figure 1B).

Module 3: Partition recurrent transfer learning (PRTL) was used to generate novel structured lead compounds for a specific target. Transfer learning can be effectively applied when there are only a few data sets available for a specific target. The molecule generation model was trained on the source domain to learn the general property and then retrained on the target domain to learn the specific property, in which the aim was to generate molecules that contain general characteristics of the source domain and specific characteristics of the target domain. The target domain can be initially divided into four subsets using the drug‐like (QED) and activity (IC_50_/pIC_50_) (IC_50_: half maximal inhibitory concentration, pIC_50_: ‐log_10_ (IC_50_)) index, respectively. Transfer learning with a high‐activity sub‐partition target domain was used as an example. The C dataset was used as the target domain and partition transfer learning (PTL; the workflow shown in Figure S1 (Supporting Information)) trained based on the VAE_FPC model parameters until the early stop condition of the PTL was reached. On the basis of this model, PTL was then carried out using the A dataset as the target domain, and the sampling procedure was subsequently performed. The present study uses PRTL to improve the novelty of the generated molecules (Figure S2, Supporting Information), which adds model parameters update on the training PTL and target domain update until the early stop condition of PRTL was reached. Novel molecules with desirable properties from the ReA dataset and ReB dataset (ReA dataset and ReB dataset are the generated novel lead compounds for PRTL with high sub‐block target domain and low sub‐block target domain, respectively) were collected and de‐duplicated for subsequent studies (Figure 1B).

Module 4: Screening and synthesizing potential novel structured lead compounds. Novelty screening was performed using the SciFinder database. Case 1: A relatively large number of target domains with a non‐definite target was obtained and the predicted pIC_50_ value of the generated novel molecules was sorted in descending order using the activity prediction model. Case 2: A small number of target domains, the docking scores of the generated novel molecules, and the dock receptor obtained via molecule docking were sorted in ascending order. The synthetic accessibility (SA) score was used to evaluate the synthetic feasibility of the molecules (SA score is calculated by a combination of fragment contributions and a complexity penalty, which ranges from 1 to 10, and the smaller the value is, the easier it is to synthesize). We selected the molecules with the lowest SA score from Top 10 and Top 11–20, respectively. Retrosynthetic analysis and route of synthesis were performed (Figure 1B).

Module 5: In vivo and in vitro drug efficacy verification experiments were performed. An in vitro cell model was used to determine the empirical IC_50_ values of the novel compounds and several known compounds were tested for comparison. Based on the characteristics of the specific disease, an in vivo animal model was established to test the efficacy of the lead compounds (Figure 1C).

### 2.2 Model Training Using the Anti‐CRC Drug Efficacy Dataset and Identification of Compound 1901

**Figure 2:**
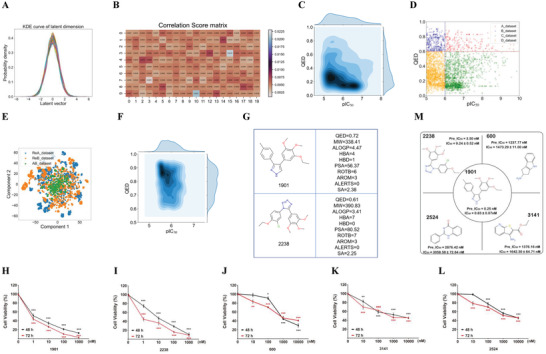
Model training using the anti‐CRC drug efficacy dataset and identification of compound 1901. A) KDE of each dimensional distribution for latent vectors for CRC molecule generation model, which was encoded by Encoder network. B) The correlation score matrix of QED property with latent vectors for CRC molecule generation model, which was obtained by FPC network, the depth of color in which indicates how important that feature is, the darker the color, the stronger the correlation. C) The joint KDE distribution of pIC_50_ and QED property for CRC dataset, the pIC_50_ value and QED value ranges in (5, 10.7) and (0.03, 0.93), respectively. D) The scatter distribution of CRC target domain, QED = 0.6 and pIC_50_ = 6 was used to divide the whole CRC target domain into four sub target domains (Adataset, Bdataset, Cdataset, Ddataset), the relationship of which can be described Methods. E) T‐SNE with ECFP4 descriptor of generate novel structured lead compounds, the resulting molecules by PRTL method form a chemical space that expands around the CRC target domain. F) The joint KDE of predicted pIC_50_ value and QED value for generate lead compounds, QED ranges in (0.60, 0.95) and predicted pIC_50_ value ranges in (5.05, 9.60). G) The physiochemical properties of compound 1901 and compound 2238. H–L) HT29 cells were co‐incubated with five compounds 1901 (H), 2238 (I), 600 (J), 3141 (K) and 2524 (L) at the indicated concentrations for 48 h or 72 h. Effect of five compounds on HT29 cell viability were measured by MTT assay and IC_50_ was calculated. N = 3 independent cell batches. One‐way ANOVA followed by Bonferroni's post hoc test for statistical analyses. M) Practical measured values of IC_50_ compared with predicted values of IC_50_ were shown. For (H–L), ^**^ *P* <0.01, ^***^ *P* <0.001 compared with the indicated group.

We initially used the data filter criteria to reprocess the ChEMBL and CRC datasets (see Experimental Section), 1 464 089 molecules were obtained and the alphabet contain 70 characters, which were applied to pretrain the VAE_FPC molecule generation model with the SMILES input form. The sampling program was then executed and the results for the CRC molecule generation model shown in Table S1 (Supporting Information). 100%, 99.84%, and 95.61% of the generated molecules were valid, unique, and satisfy the drug‐like property. This molecule generation model has a better generative ability using comparative studies.^[^ ^8^, ^15^, ^16^, ^17^ ^]^ The kernel density estimate (KDE) of the latent vectors when encoding 1600 molecules randomly selected from the training dataset for CRC, as shown in **Figure** **2**A, which showed that each dimension of the latent vectors obeys a normal distribution. The output of the FPC network was the correlation score matrix (it should be 200 × 1, but was reshaped to 20 × 10 for simplicity), which is the relationship between the latent vectors and condition property (i.e., the QED drug‐like index) obtained using the batch size molecules randomly selected training dataset of the CRC molecule generation model, as shown in Figure 2B. This shows that the importance between the different dimensions of the latent vectors and condition property was different, the darker the color, the stronger the correlation. These results highlight the FPC network can capture the relationship between molecule condition property and latent space.

Subsequently, the VAE_FPC molecule generation model was retrained using PTL (a comparison experiment of the optimal impatience for the different CRC subsets shown in Figure S3 (Supporting Information)) with the CRC target domain (for details, see Experimental Section; the joint KDE distribution and scatter distribution for the CRC target domain is shown in Figure 2C,D, respectively) to generate molecules that exhibit pharmacological activity. The drug efficacy classification model was trained using the CRC dataset to evaluate whether the generated novel molecules have pharmacological activity for CRC. We evaluated the model performance of different molecule representations coupled with several modeling methods, including Avalon, ECFP4, Rdkit molecule fingerprints combined with SVM, RF, and GBDT modeling methods. The comparative results show that the optimal model performance was observed using Avalon_GBDT as the CRC activity classification model. The accuracy and F1‐score of test set were 84.79% and 0.858, respectively. (CRCACM; the comparative results are shown in Tables S2–S4 (Supporting Information) for CRCACM).

Finally, this paper proposes PRTL to improve the novelty of the generated molecules (the comparative results are shown in Tables S5–S8 (Supporting Information)). In the cycle, only the novel molecules generated with the desired properties (not included in the training dataset and finetune dataset) were collected to update the PTL target domain and model parameters until the early stop condition of the PRTL was reached, resulting in a total of 3346 de novo design molecules that satisfy the chemically valid, drug‐like, and pharmacological activity requirements for CRC. Analysis of the chemical space in which the molecules with the ECFP4 fingerprint using T‐SNE shows that the resulting molecules cannot only cover the molecules in the target domain, but also expand the new chemical space (T‐SNE of the target domain (green)) and generate molecules (blue and orange) for CRC, as shown in Figure 2E. The joint KDE distribution of the predicted pIC_50_ values (predicted using CRCAPM and the comparative results of CRCAPM shown in Tables S9–S11 (Supporting Information)), the MRE, MAE, RMSE of training set was 1.30%, 0.08, 0.222, the MRE, MAE, RMSE of test set was 6.86%, 0.42, 0.644, respectively) and QED values of the generated molecules for CRC are shown in Figure 2F. When compared with CRC target domain (Figure 2C), the properties of the generated molecules were constantly optimized, especially for the QED property, and the predicted pIC_50_ values range from 5.05 to 9.60. These results indicate that the PRTL can generate novel molecules with desirable properties for CRC.

A novelty check was performed using the SciFinder database. For the CRC study, the number of novel and existing molecules was 3128 and 218, respectively. CRCAPM was used to predict the pIC_50_ values and sort the results in descending order. The SMILES of Top20 and their corresponding properties are shown in Table S23 (Supporting Information). Two compounds (the physicochemical and structural properties, and molecule structure are shown in Figure 2G) with the lowest SA score (1901 and 2238) were selected from the Top 10 and Top 11–20, respectively. The corresponding similarity distribution with the molecules of CRC target domain is shown in Figure S5A,B (Supporting Information).

Novel structured compounds 1901 and 2238 were synthesized. Three known compounds (600, 3141, and 2524) were studied for comparison. HT29 cells were co‐incubated with each compound at the specified concentrations for 48 or 72 h (Figure 2H–L). Their effects on the HT29 cell viability were measured using the MTT assay and the IC_50_ values calculated. The results in Figure 2M, suggested that the IC_50_ values measured experimentally were compared with the predicted values (Figure 2M). Among these compounds, the experimental IC_50_ values obtained for compounds 1901, 2238, 600, 3141, and 2524 were close to their predicted values, indicating the precision of this model (CRCAPM).

Compounds 1901 and 2238 exhibit a potent inhibitory effect on the HT29 cell viability with IC_50_ values of 0.83 and 8.25 nm, respectively. Compound 1901 showed a better effect and thus was further evaluated in vivo.

### 2.3 In vivo Efficacy Investigation of Compound 1901 Against CRC

**Figure 3:**
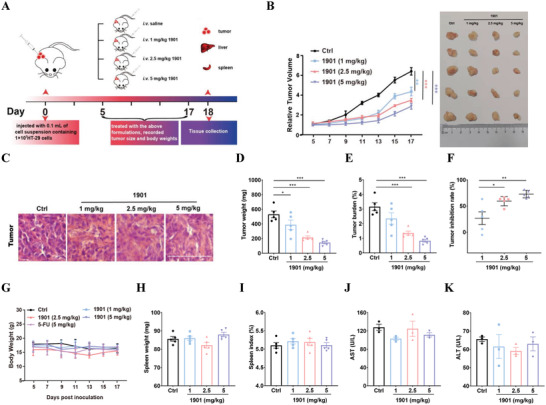
In vivo efficacy investigation of compound 1901 against CRC. A) Experiment design for anti‐CRC efficacy exploration of compound 1901. B) The effect of compound 1901 on tumorigenesis were analyzed. Tumor size were monitored during the administration of compound 1901. Pictures of tumor were shown. C) Representative pictures of HE staining for tumor tissue were shown. D–F) Tumor weight, tumor burden and tumor inhibition rate were calculated. G) The body weight of HT29 bearing nude mice were monitored during the administration of compound 1901. H,I) spleen weight and spleen index were calculated for immune organ analysis. J,K) ALT and AST were measured using commercial kits for liver function analysis. N = 5 different animals. One‐way ANOVA followed by Bonferroni's post hoc test for statistical analyses. For (B,D–F), ^*^ *P* < 0.05, ^**^ *P* < 0.01, ^***^ *P* < 0.001 compared with the indicated group.

A subcutaneous xenograft model was established to study the effect of compound 1901 on CRC in vivo, and the experiment design shown in **Figure** **3**A. The tumor size and animal body weight were monitored during the administration of compound 1901. The results in Figure 3B show that compared to the control group, compound 1901 inhibits tumor growth (day 17) (1 mg kg^−1^: *p* < 0.01, 2.5 mg kg^−1^: *p* < 0.001, 5 mg kg^−1^: *p* < 0.001). Hematoxylin and eosin (HE) staining showed that compared to the control group, treatment with compound 1901 had an obvious effect on tumor suppression in Figure 3C. Moreover, upon the administration of compound 1901, the tumor weight (1 mg kg^−1^: *p* < 0.05, 2.5 mg kg^−1^: *p* < 0.001, 5 mg kg^−1^: *p* < 0.001) and tumor burden (2.5 mg kg^−1^: *p* < 0.001, 5 mg kg^−1^: *p* < 0.001) significantly decreased compared to the control group (Figure 3D,E). Tumor inhibition rate increased dose‐dependently (Figure 3F). The results show the weight of the mice among the different groups displayed no significant difference (Figure 3G). The spleen weight and spleen index were calculated for immune organ analysis (Figure 3H,I). Alanine transaminase (ALT) and aspartate transaminase (AST) were measured using commercial liver function analysis kits (Figure 3J–K). Compound 1901 showed no obvious toxicity or side effects on the spleen and liver. Our results indicate that compound 1901 exhibits anti‐CRC activity.

### 2.4 Insights into the Mechanisms of Compound 1901 on CRC

**Figure 4:**
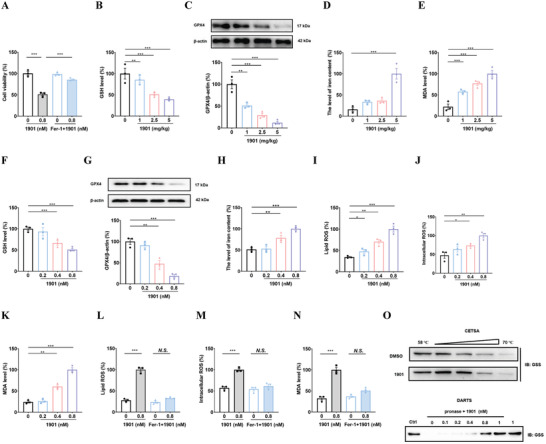
Insights into the mechanisms of compound 1901 on CRC. A) The effect of compound 1901 on cell viability was measured combined with ferroptosis inhibitor Fer‐1 (10 µm, 72 h). N = 3 independent cell batches. B–E) The effects of 1901 on ferroptosis in tumor tissue were analyzed. N = 3 different animals. F–K) The effects of compound 1901 on ferroptosis in HT29 cells were analyzed. N = 3 independent cell batches. GSH content, free iron level and MDA level were measured using commercial kits. GPX4 protein were measured by western blot. Lipid ROS was measured using BODIPY 581/591 C11 staining. ROS level was tested using dihydroethidium staining. L–N) The effect of compound 1901 on ROS, lipid ROS and MDA levels were analysis combined with ferroptosis inhibitor Fer‐1 (10 µm, 72 h). N = 3 independent cell batches. O) Compound 1901 promoted resistance of GSS to different temperature gradients by CETSA and compound 1901 promoted resistance of GSS to proteases by DARTS. N = 3 independent cell batches. One‐way ANOVA followed by Bonferroni's post hoc test for statistical analyses for (B–K), Two‐way ANOVA followed by Bonferroni's post hoc test for statistical analyses for (A,L–N). ^*^ *P* < 0.05, ^**^ *P* < 0.01, ^***^ *P* < 0.001 compared with the indicated group.

On the basis of drug efficacy, the mechanism of compound 1901 on cell death pathway was further explored by an RNA‐seq assay. Upon treatment with compound 1901, different expressed genes were enriched in oxidative phosphorylation and ferroptosis (Figure S6A, Supporting Information). Oxidative phosphorylation regulates oxidative cell death and ferroptosis is a unique iron‐dependent form of oxidative cell death. Ferroptosis‐related genes overlap with canonical antioxidant pathway. **Figure** **4**A shows Ferrostatin‐1 (Fer‐1), a well‐known ferroptosis inhibitor, alleviated compound 1901‐induced cell death (1901/control: *p* < 0.001, 1901/Fer‐1+1901: *p* < 0.001). The effect of compound 1901 on other cell death pathways were also examined. As shown in Figure S6B (Supporting Information), the apoptosis inhibitor Z‐VAD‐fmk, autophagy inhibitor 3‐MA or pyroptosis inhibitor disulfiram could not completely rescue compound 1901‐induced cell death. These results suggested that the anti‐CRC effect of compound 1901 may largely depend on its ferroptosis promoting effect. Ferroptosis can be triggered via a depletion in glutathione (GSH). Glutathione peroxidase 4 (GPX4) competes with lipid peroxidation by oxidizing GSH, and the activation of GPX4 is highly dependent on intracellular GSH. Iron overload is the main feature of ferroptosis, accompanied by the accumulation of reactive oxygen species (ROS) and lipid peroxidation products. Figure 4B–E shows that compared to the control group, compound 1901 can decrease the GSH content (1 mg kg^−1^: *p* < 0.01, 2.5 mg kg^−1^: *p* < 0.001, 5 mg kg^−1^: *p* < 0.001) and the expression of GPX4 (1 mg kg^−1^: *p* < 0.01, 2.5 mg kg^−1^: *p* < 0.001, 5 mg kg^−1^: *p* < 0.001), and increase the levels of free iron (5 mg kg^−1^: *p* < 0.001) and MDA (1 mg kg^−1^: *p* < 0.001, 2.5 mg kg^−1^: *p* < 0.001, 5 mg kg^−1^: *p* < 0.001) in vivo. Moreover, compared to the control group, compound 1901 can decrease the GSH content (0.4 nm: *p* < 0.001, 0.8 nm: *p* < 0.001) and GPX4 expression (0.4 nm: *p* < 0.01, 0.8 nm: *p* < 0.01), and increase the level of free iron (0.4 nm: *p* < 0.01, 0.8 nm: *p* < 0.001), lipid ROS (0.2 nm: *p* < 0.05, 0.4 nm: *p* < 0.01, 0.8 nm: *p* < 0.001), intracellular ROS (0.4 nm: *p* < 0.05, 0.8 nm: *p* < 0.001), and MDA level (0.4 nm: *p* < 0.01, 0.8 nm: *p* < 0.001) in vitro (Figure 4F–K). Figure 4L–N shows Fer‐1 eliminated the compound 1901‐induced changes in the lipid ROS (1901/control: *p* < 0.001), intracellular ROS (1901/control: *p* < 0.001), and MDA level (1901/control: *p* < 0.001). CETSA (cellular thermal shift assay) was performed to explore the target protein for compound 1901, and the target protein was identified as glutathione synthetase (GSS) using LC‐MS/MS analysis (Figure S6C, Supporting Information). GSS is the cytosolic enzyme that catalyzes GSH biosynthesis. Molecular docking showed the binding mode between compound 1901 and GSS (Figure S6D, Supporting Information). Furthermore, the CETSA and DARTS (drug affinity responsive target stability) results confirm that GSS was the target protein of compound 1901 (Figure 4O). These results indicate that triggering ferroptosis by compound 1901 may play an important role in CRC treatment.

### 2.5 Model Training Using the Anti‐AD Drug Efficacy Dataset and Identification of Compound 548

**Figure 5:**
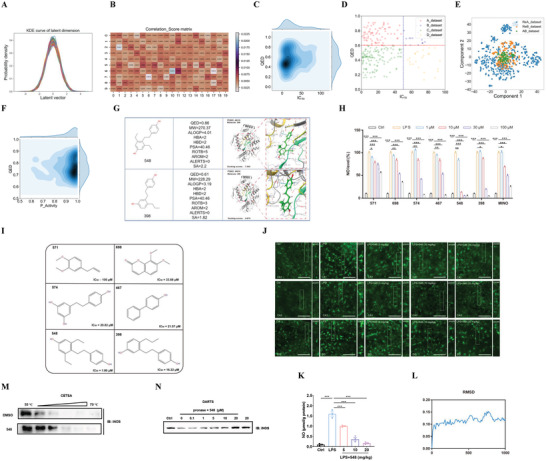
Model training using the anti‐AD drug efficacy dataset and identification of compound 548. A) KDE of each dimensional distribution for latent vectors for AD molecule generation model, which was encoded by Encoder network. B) The correlation score matrix of QED property with latent vectors for AD molecule generation model, which was obtained by FPC network, the depth of color in which indicates how important that feature is, the darker the color, the stronger the correlation. C) The joint KDE distribution of IC_50_ and QED property for AD dataset, the IC_50_ value and QED value ranges in (4, 7) and (0.13, 0.91), respectively. D) The scatter distribution of AD target domain, QED = 0.6 and IC_50_ = 50 was used to divide the whole AD target domain into four sub target domains. E) T‐SNE with ECFP4 descriptor of generate novel structured lead compounds for AD, the resulting molecules by PRTL method form a chemical space that expands around the AD target domain. F) The joint KDE distribution of predicted activity probability value and QED value for generate molecules, QED ranges in (0.60, 0.94) and the activity probability values were all higher than 0.58. G) Physiochemical properties and docking scores between compound 548 and iNOS, and compound 398 and iNOS. H) BV‐2 cells were co‐incubated with six compounds at the indicated concentrations for 24 h. Effect of six compounds (548, 398, 571, 698, 574 and 467) on NO release were measured by nitrite assay. N = 3 independent cell batches. One‐way ANOVA followed by Bonferroni's post hoc test for statistical analyses. I) chemical structure of the compounds and calculated IC_50_. J,K) LPS induced mice model was established. Iba‐1 positive cells by immunofluorescence in CA1, CA3 and DG of brain tissue were shown (J). NO content by nitrite assay in brain tissue was measured after different dose of 548 (5, 10, 20 mg kg^−1^) treatment (K). N = 3 different animals. One‐way ANOVA followed by Bonferroni's post hoc test for statistical analyses for (K). L) RMSD curve of compound 548 and iNOS. M) Compound 548 promoted resistance of iNOS to proteases by DARTS and compound 548 promoted resistance of iNOS to different temperature gradients by CETSA. N = 3 independent cell batches. For (H) and (K), ^*^ *P* < 0.05, ^**^ *P* < 0.01, ^***^ *P* < 0.001 compared with the indicated group.

We initially used the data filter criteria to reprocess the ChEMBL and AD datasets. 1 464 761 molecules and 69 characters were obtained. The sizes of the training dataset and alphabet were slightly different to those used for CRC due to the different molecules in the target domain (see Experimental Section), which were applied to pretrain the VAE_FPC molecule generation model with SMILES used as the input. The sampling program was then executed and the sampling results for the AD molecule generation model shown in Table S12 (Supporting Information), which indicate that the VAE_FPC molecule generation model can be used to generate chemically valid and drug‐like molecules. The KDE of the latent vectors show that each dimension of the latent vectors obeys a normal distribution (**Figure** **5**A). The correlation score matrix was used as the output of the FPC network, as shown in Figure 5B, which further verified the importance between different dimensions of the latent vectors and condition property was different.

Subsequently, the VAE_FPC model was retrained using the PTL with the AD target domain (the joint KDE distribution and scatter distribution for the AD dataset is shown in Figure 5C,D, and the comparison experiment of the optimal impatience for the different AD subsets is shown in Figure S4 (Supporting Information)). To evaluate whether the generated novel molecules have pharmacological activity for AD, the drug efficacy classification model was trained on the AD dataset. We assessed the model performance for Avalon, ECFP4, Rdkit molecule fingerprint coupled with SVM, RF, GBDT modeling methods and MI, and Lasso feature selection method. Upon comparing the results, the optimal model performance was observed when using the Lasso_Rdkit_SVM model as the AD activity classification model. The accuracy and F1‐score of test set of ADACM was 98.32% and 0.983, respectively (ADACM; the results are shown in Tables S13–S18 (Supporting Information)).

PRTL was used to improve the novelty of the generated molecules (the comparative results are shown in Tables S19–S22 (Supporting Information)), resulting in a total of 747 de novo designed molecules that satisfy the chemically valid, drug‐like, and pharmacologically activity requirements for AD. Analysis of the chemical space shows that the resulting molecule can not only cover the molecules in the AD target domain, but also expand new chemical space (T‐SNE of the target domain (green) and generate molecules (blue and orange) for AD, as shown in Figure 5E). The joint KDE distribution of the predicted activity probability and QED values for AD are shown in Figure 5F. Compared with the target domain (Figure 5C), the QED property of the generated molecules were constantly optimized. These results indicate that the PRTL can generate novel molecules with desirable properties for AD.

A novelty check was performed using the SciFinder database. For AD, the number of novel and known molecules obtained was 556 and 191, respectively. Molecule docking was used to predict the docking scores, which were sorted in ascending order. The SMILES of Top20 and their corresponding properties are shown in Table S24 (Supporting Information). Compounds 548 and 398 (the corresponding similarity distribution with AD target domain are shown in Figure S5C,D (Supporting Information)) were selected from Top 10 and Top 11–20 with the lowest SA score, whose physicochemical and structural properties are shown in Figure 5G, exhibit a 2D diagram showing the interaction between the ligand (small molecules) and iNOS (PDB ID: 4NOS), and docking scores of −7.049 and −6.679, respectively.

The novel structured compounds (548 and 398) were synthesized, and several known compounds (571, 698, 574, and 467) were studied for comparison. Figure 5H and I shows the effect of each compound on the LPS‐induced release of Nitric Oxide (NO) in activated microglia was investigated and the IC_50_ value calculated. Compound 548 shows the most potent activity toward NO release, which was consistent with the predicted results. Furthermore, the inhibitory effect of compound 548 on microglial activation and the NO level were investigated in vivo. Figure 5J shows compound 548 (5, 10, 20 mg kg^−1^) inhibited the number of Iba‐1 positive cells in LPS‐induced mice in the hippocampus CA1, CA3, and DG regions. Moreover, Figure 5K shows that compared to LPS group, compound 548 (5 mg kg^−1^: *p* < 0.001, 10 mg kg^−1^: *p* < 0.001, 20 mg kg^−1^: *p* < 0.001) significantly inhibited the NO level. The IC_50_ value of compound 548 inhibiting NO in this in vivo animal model was ≈5 mg kg^−1^ and therefore this dose was chosen as the highest dose in the following in vivo animal model. Molecular simulations showed the RMSD curve of compound 548 and iNOS in Figure 5L, suggesting the stable binding mode between iNOS and compound 548. CETSA and DARTS were then performed to explore its mechanism of action. Figure 5M shows that iNOS was gradually degraded with an increase in temperature and the expression of iNOS decreased. However, the stability of iNOS was enhanced after treatment with compound 548. Moreover, Figure 5M shows iNOS was degraded in the presence of protease and the stability of iNOS was enhanced upon increasing the concentration of compound 548.

### 2.6 Efficacy Investigation of Compound 548 against AD

**Figure 6:**
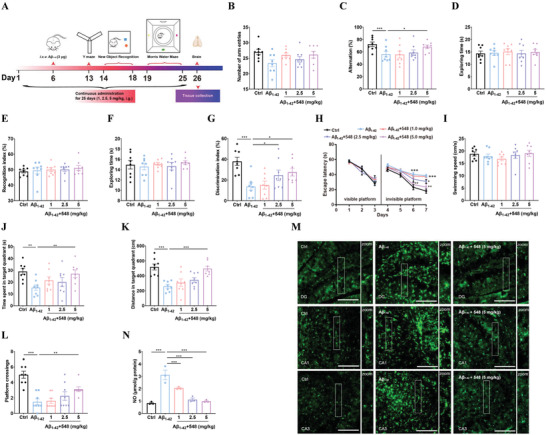
Efficacy investigation of compound 548 against AD. A) Aβ_1‐42_‐induced AD model was established and the experiment schedule of behavioral assessment. B,C) Y‐maze test was used to measure working memory impairment of Aβ_1‐42_‐treated mice. Number of arm entries (B) and Alternation (C) were measured. N = 8 different animals. D–G) novel object recognition task was used to measure visual recognition ability of Aβ_1‐42_‐treated mice. Exploring time (D) and Recognition index (E) in the acquisition stage, Exploring time (F) and Discrimination index (G) in the test stage were measures. N = 8 different animals. H–L) Morris water maze test was used to measure spatial learning and memory impairment of Aβ_1‐42_‐treated mice. Escape latency (H), Swimming speed (I), Time spent in target quadrant (J), Distance spent in target quadrant (K) and Platform crossings (L) were measures. N = 8 different animals. M) Iba‐1 positive cells by immunofluorescence in CA1, CA3 and DG of brain tissue were shown. N) NO content by nitrite assay in brain tissue was measured after compound 548 treatment. N = 3 different animals. One‐way ANOVA followed by Bonferroni's post hoc test for statistical analyses. ^*^ *P* < 0.05, ^**^ *P* < 0.01, ^***^ *P* < 0.001 compared with the indicated group.

An Aβ_1‐42_‐induced AD animal model was established to investigate the anti‐AD activity of compound 548 based on its inhibitory effect on neuroinflammation. Cognitive impairment is one of the the main features of AD. A Y‐maze test was carried out to analyze the working memory in mice. **Figure** **6**B shows no significant differences were observed in the total number of arm entries among the groups, revealing that the locomotor activity of mice was not affected. Figure 6C shows that compared to the control group, the spontaneous alternation significantly decreases in the Aβ_1‐42_‐treated mice (*p* < 0.001), and compound 5 mg kg^−1^ 548 (*p* < 0.05) significantly increased spontaneous alternation. The results suggest that compound 548 attenuated working memory impairment. The novel object recognition task was carried out in order to analyze the visual recognition ability in mice. Figure 6D and E show that mice spent a similar time to explore objects and did not show any preference for either of the identical objects among groups in the acquisition stage. In the test stage, mice spent similar total time to explore objects as shown in Figure 6F. However, Figure 6G shows compared to the control group, the discrimination index significantly decreases in the Aβ_1‐42_‐treated mice (*p* < 0.001), and compound 548 (2.5 mg kg^−1^: *p* < 0.05, 5 mg kg^−1^: *p* < 0.05) significantly increased the discrimination index. The results suggest compound 548 ameliorates the visual recognition ability. A Morris water maze test was used to analyze the spatial learning and memory ability in mice. Figure 6H shows no significant differences were observed in the escape latency among the groups in the visible platform stage. On the sixth day of the invisible platform stage, compared to the control group the escape latency increases in the Aβ_1‐42_‐treated mice (*p* < 0.001), and compound 548 (5 mg kg^−1^: *p* < 0.01) decreased the escape latency on the sixth day. On the seventh day of the invisible platform stage, compared to the control group the escape latency increases in the Aβ_1‐42_ treated mice (*p* < 0.001), compound 548 (1 mg kg^−1^: *p* < 0.05, 2.5 mg kg^−1^: *p* < 0.01, 5 mg kg^−1^: *p* < 0.001) decreased the escape latency on the seventh day. In the invisible platform stage, no significant differences were observed in the swimming speed among the groups, as shown in Figure 6I. Compared to the control group, the swimming time (*p* < 0.01) and swimming distance (*p* < 0.001) in the target quadrant, and platform crossings numbers (*p* < 0.001) significantly decrease in the Aβ_1‐42_‐treated mice, while compound 548 significantly increased the swimming time (5 mg kg^−1^: *p* < 0.01) and swimming distance (5 mg kg^−1^: *p* < 0.001) in the target quadrant, and platform crossings numbers (Figure 6J–L). The results suggest compound 548 decreased spatial learning and memory impairment. The effect of compound 548 on the NO level and microglial activation in Aβ_1‐42_‐treated mice was further investigated. Figure 6N shows compared to the control group, the NO level increases in Aβ_1‐42_‐treated mice (*p* < 0.001), and compound 548 (1 mg kg^−1^: *p* < 0.001, 2.5 mg kg^−1^: *p* < 0.001, 5 mg kg^−1^: *p* < 0.001) significantly decreased the NO level. The number of Iba‐1 positive cells increases in Aβ_1‐42_‐treated mice, and compound 548 (5 mg kg^−1^) significantly decreased the number of Iba‐1 positive cells in the hippocampus CA1, CA3, and DG region, as shown in Figure 6M. Our developed strategy has also been successfully used for the discovery of a novel structured anti‐AD drug.

## 3 Discussion

A major difficulty in traditional drug development is finding novel chemical compounds with desirable drug efficacy. To overcome this difficulty, we have developed a DTLS, which realizes the generation and identification of novel compounds with drug efficacy. In our study, the DTLS was trained using a disease‐direct‐related activity dataset combined with a computational model considering the drug‐like properties, novelty, activity, and synthetic feasibility. In addition, we coupled our DTLS with both in vitro and in vivo studies and mechanism exploration as a comprehensive workflow, which allows novel compounds to be rapidly discovered, synthesized, and verified using in vitro and in vivo studies, and the mechanisms of the novel compounds revealed.

The DTLS is capable of providing a new approach to simplify the traditional drug development process. As is known, drug development is a long and complex process that includes target selection, lead compound identification, preclinical study, and clinical trials, among which the inefficient discovery of early lead compounds is an important issue that needs to be urgently resolved. Traditionally, a mass of hit compounds can be obtained from natural products, molecular libraries, and high‐throughput screening, followed by intensive structural optimization, synthesis and validation steps, which are time‐consuming and expensive, and it is not always possible to discover a lead compound.^[^ ^18^ ^]^ The DTLS exhibits the advantage of directly generating molecules with the desired drug efficacy by using a disease‐direct‐related activity dataset as the input, which improves the efficiency of lead compound discovery.

Currently, most machine learning algorithms applied to drug design are based on protein targets.^[^ ^8^, ^10^, ^11^, ^16^, ^19^ ^]^ For example, Francesca Grisoni proposed modular design‐make‐test‐ analyze platform to design LXR agonists.^[^ ^8^ ^]^ Zhavoronkov proposed the generative tensorial reinforcement learning method based on deep learning for the design of small molecules and discovery of potent inhibitors of DDR1.^[^ ^10^ ^]^ M. Popova proposed the reinforcement learning for structural evolution to develop novel putative inhibitors of JAK2.^[^ ^11^ ^]^ Marcus Olivecrona proposed reinvent model to generate compounds predicted to be active against DRD2.^[^ ^16^ ^]^ Li group proposed a generative deep learning model and applied it to the discovery of a potent and selective inhibitor of RIPK1.^[^ ^19^ ^]^ These successful cases demonstrate the effectiveness of target‐based de novo design based on deep learning. However, these approaches cannot address many diseases without a clear target protein.^[^ ^14^ ^]^ In contrast, the application of our DTLS is independent of the disease‐related target protein, providing the broader usability of machine learning in the drug discovery process. In our study, the DTLS was first applied toward the design of anti‐cancer drugs and we collected a training dataset of compounds with potential efficacy regardless of the pathogenic target proteins. The training dataset contains compounds with an inhibitory effect on HT29 cell proliferation, and the DTLS was then performed to generate novel structured molecules. When combined with a drug efficacy‐based screening strategy, compound 1901 was discovered. Currently, most research studies focus on the application of AI theory or algorithms for molecule design and few have conducted drug efficacy verification studies. In contrast, we coupled the DTLS with both in vitro and in vivo validation and mechanism exploration studies as a comprehensive workflow to provide an overall evaluation of the proposed strategy. Thus, compound 1901 was synthesized and tested using in vitro and in vivo experiments. Meanwhile, the validity of the CRCAPM used in this study was verified by in vitro studies on the two novel structured compounds and three known compounds. This indicates that CRCAPM may be used to screen anti‐CRC compounds from the public database in future research studies. RNA‐seq was then performed, the differently expressed genes were analyzed and revealed that the anti‐CRC effect of compound 1901 may depend on its regulation on oxidative cell death, and triggering ferroptosis by compound 1901 may plays an important role in CRC treatment. Ferroptosis can be induced by inhibiting the biosynthesis of GSH.^[^ ^20^, ^21^ ^]^ Upon searching for the protein targets of compound 1901, we found that compound 1901 can bind with GSS, which is the cytosolic enzyme catalyzing GSH biosynthesis. Our findings suggest that the anti‐CRC effect of compound 1901 may depend on its binding with GSS. In this case, target exploration was performed in a later stage, rather than at the very beginning in the traditional way, which provides a new option in the drug discovery pattern. The above‐mentioned DTLS‐in vitro and in vivo validation‐mechanism exploration workflow has been proven effective toward generating and identifying a novel compound with drug efficacy for CRC and revealed the mechanism of the novel compound. This suggests that the DTLS is has great potential to find novel lead compounds with drug efficacy for a particular disease without definite target proteins, which provides an effective new strategy for drug discovery.

The DTLS was also successfully applied to AD. In this case, our DTLS coupled with target‐based screening was applied, which is different for the former. AD belongs to a class of diseases with complex pathogenesis and is difficult to cure. Aβ and Tau have long been considered as the representative pathological changes observed in AD. However, clinical drug candidate developments targeting Aβ and Tau have struggled to produce positive results.^[^ ^7^ ^]^ Recently, it has been reported that microglia activation‐induced neuroinflammation is more prevalent in older adults and is more pronounced in patients with cognitive impairment and AD‐related dementia,^[^ ^22^ ^]^ indicating that activated microglia‐mediated neuroinflammation may play a crucial role in the occurrence and development of AD. In addition, some research studies have demonstrated that microglia‐targeted therapies in AD models also achieve satisfactory results,^[^ ^23^, ^24^, ^25^, ^26^, ^27^ ^]^ suggesting that inhibiting microglial activation may be a promising strategy to develop anti‐AD drugs. The excessive release of NO is considered to be the hallmark of microglial activation and involved in contributing to the development of AD.^[^ ^28^ ^]^ As reported, compounds with the capacity of inhibiting microglial activation and the excessive release of NO significantly improve the cognitive outcome in AD animal models.^[^ ^29^, ^30^ ^]^ Therefore, the inhibition of NO release in microglia is suitable to serve as disease‐direct‐related activity in AD. Different from the former case, this disease‐direct‐related activity was coupled with a target protein (iNOS), since NO is synthesized from l‐arginine by iNOS in microglia.^[^ ^28^ ^]^ This feature was fully utilized in the subsequent screening stage. In this case, the DTLS was proven to be effective in generating novel structured compounds with potential anti‐AD activity. The training dataset contains compounds with an inhibitory effect on NO release in microglia. After the generation of novel structured molecules combined with a target‐based screening strategy, followed by in vitro and in vivo experiments, we found that compound 548 exhibited a potential anti‐AD effect. Furthermore, CETSA and DARTS were conducted and revealed that iNOS may act as the target protein for compound 548. Therefore, our DTLS can also use a disease‐direct‐related activity dataset, either associated with a certain target or independent of the target protein, as the input. In the former case, a target‐based screening strategy can be utilized and the dataset is not necessarily too large. In addition, it has the advantage of facilitating the mechanistic study of the novel lead compounds in the follow‐up mechanism exploration stage. In this case of AD, the target protein was involved in the relative former stage, which was different from that used in the case of CRC, and indicates a more flexible exploration or use of the target. The above‐mentioned CRC and AD cases have revealed that the DTLS is effective for generating novel structured molecules with drug efficacy, which provides a powerful and promising approach for diseases with complex pathogenesis or undefined targets. The DTLS may also have a wider range of applications, for example, multi‐target screening can be performed during the screening stage and novel compounds with multiple targets may be identified.

Although the molecules are designed using machine‐learning algorithms, it remains to be seen whether these molecules actually exert their corresponding drug efficacy in vitro and in vivo. Herein, we established an DTLS integrated VAE_FPC network and PRTL. The VAE_FPC network was trained by reprocessing the ChEMBL dataset to generate chemically valid and drug‐like molecules. Using comparative experiments, it can be verified that the proposed FPC network can increase the proportion of generated molecules satisfying the drug‐like property requirement. On this basis, the PRTL was trained using the disease‐direct‐related activity dataset to generate novel chemical compounds with desirable drug efficacy. The PRTL proposed in this study can further improve the efficiency and novelty of generating molecules with desirable biological properties. The successful application of our DTLS was verified using in vivo and in vitro experiments. Our results have shown that the DTLS was well‐suited to generate molecules with the desired biological properties. In addition, accurate activity ranking of the generated molecules is a key prerequisite for subsequent research studies. In this paper, we first proposed the CRCAPM model for sorting. The results of our experiment showed that the compound selected from the Top 10 and Top 11–20 exerted better drug efficacy than the compounds selected from the bottom list.

In the present study, two types of disease models were carefully selected. Cancer is one of the leading causes of mortality in the world. It is reported that dozens of drug candidates with potential to treat various tumors enter clinical trials each year, but fewer than 4% will ultimately obtain approval by the FDA.^[^ ^31^ ^]^ Although various factors are associated with this failure, a major cause is the incomplete understanding of the complex pathogenesis and multiple targets of cancer. Currently, many research studies have focused on anti‐cancer agents and a large amount of experimental data can be collected as the training dataset, among which the data involving compounds exerting an inhibitory effect on CRC cell proliferation is extensive. Moreover, there are no effective therapeutic targets for CRC, which is consistent with the input settings of the model. AD is a highly‐prevalent and progressive neurodegenerative disorder, which is the main cause of dementia. It has been reported that drug candidates in AD clinical trials have an estimated failure rate of 99.6%.^[^ ^32^, ^33^ ^]^ Currently, only four drugs have been approved as treatments for AD, which can only control or delay the disease, rather than cure it. Therefore, new anti‐AD drugs are urgently needed. Due to the relatively few measured data that can be collected, only 238 compounds that inhibits NO release in microglia were gathered. Based on the training dataset, we have also discovered an effective anti‐AD drug, further demonstrating the universality of the DTLS for both large and small training datasets. These cases suggested that it is possible for our model to be used for de novo generation of compounds for any target of any disease under the condition that a certain number of activity molecules can be obtained as input training datasets to establish accurate activity prediction model. However, for some diseases, it is difficult to collect a large amount of activity data. Considering this, quantitative and qualitative activity prediction models were established based on the quantity of activity data. For diseases with larger active data sets, quantitative activity prediction model is applicable. When the training data set is too small to establish a more accurate quantitative activity prediction model, the qualitative activity prediction model is applicable. In this case, other activity relevant information is needed to screen the generated molecules. Meanwhile, our results suggested that activity relevant information‐based screening strategy also have the advantage for the following‐up mechanism research.

In summary, based on DTLS‐in vitro and in vivo validation‐mechanism exploration workflow, we discovered the novel structured lead compounds 1901 and 548, which had anti‐CRC and anti‐AD activity, respectively. DTLS could not only realize the generation and identification of novel compounds with drug efficacy, but also had the advantage of identifying compounds by focusing on protein targets to facilitate the mechanism study. This work highlights the significant impact of machine learning on the design of novel compounds with drug efficacy, which provides a powerful new approach to drug discovery.

## 4 Experimental Section

### Training Dataset and Finetune Dataset

The ChEMBL (https://www.ebi.ac.uk/chembl/) database was reprocessed for the training VAE_FPC procedure by using the following data filtering criteria: Canonical SMILES should be successfully parsed using rdkit software (http://www.rdkit.org/) and the molecule with a maximum length does not exceed 120 characters; the number of heavy atoms is in the range of 5–70; atomic number ∈ [0, 6, 7, 8, 9, 16, 17, 35]; remove active molecules that have inhibitory effect on the disease. 1 464 089 molecules were retained by the above pretreatment process. The alphabet was constructed by using 1 464 089 molecules and the obtained active molecules, which contained 70 characters for CRC molecule generation model. For AD molecule generation model, 1 464 761 molecules were retained by the above pretreatment process. The alphabet was constructed similarly by using 1 464 761 molecules and the obtained active molecules, the size of alphabet was 69 characters. For comparative experimental study, the above data set was further screened for QED higher than 0.6 to train molecule generation model. For CRC molecule generation model 697 748 molecules and 58 characters were used, for AD molecule generation model 697 912 molecules and 57 characters were used.

The IC_50_ value was used to obtain active data sets. For CRC dataset, the small molecules were downloaded from the ChEMBL database. Briefly, the IC_50_ data of HT29 cells and the target type was set as cell line were searched. Further, the data of 14 077 small molecules were downloaded through screening. Assay Description was taken as the screening standard and the data obtained from MTT experiment were selected and a total of 6277 molecules were screened (3261 molecules as target domain that was biological activity and satisfy the above data filtering criteria). For AD dataset, if the IC_50_ value is less than 100 µm, it was classified into active data sets, which obtained from the experimental data. Dataset was divided by QED and IC_50_ index as follows:

(1) $$\begin{array}{ccc} & & \left\{\begin{array}{c} A_{dataset}:Drug-like\;and\;High\;activity\;\;\;\;\;\;\left(QED>\partial_{QED}\;and\;IC_{50\min}\mu M<IC_{50}<\partial_{IC50}\mu M\right)\\ B_{dataset}:Drug-like\;and\;Low\;activity\;\;\;\;\;\;\;\left(QED>\partial_{QED}\;and\;\partial_{IC50}\mu M\leq IC_{50}\leq IC_{50\max}\mu M\right)\\ C_{dataset}:Non\;drug-like\;and\;High\;activity\left(QED\leq \partial_{QED}\;and\;IC_{50\min}\mu M<IC_{50}<\partial_{IC50}\mu M\right)\;\\ D_{dataset}:Non\;drug-like\;and\;Low\;activity\;\left(QED\leq \partial_{QED}\;and\;\partial_{IC50}\mu M\leq IC_{50}\leq IC_{50\max}\mu M\right) \end{array}\right.\\ & & \;\partial_{QED}=0.6,\;IC_{50\min}=0\;\;\mathrm{in}\;\mathrm{this}\;\mathrm{study},\;\partial_{IC50}=1,\;IC_{50\max}=10\;\mathrm{for}\;\mathrm{the}\;\mathrm{CRC}\;\mathrm{dataset},\;\\ & & \;\partial_{IC50}=50,\;IC_{50\max}=100\;\mathrm{for}\;\mathrm{the}\;\mathrm{AD}\;\mathrm{dataset} \end{array}$$

### Molecule Generation Model

The VAE_FPC network was trained to generate chemically valid and drug‐like molecules using training dataset. It includes three parts: Encoder network, FPC network, Decoder network (Figure 1A). The main function of Encoder network was to convert the input molecules into latent vectors with continuous fixed‐dimension. Training molecules were represented by SMILES form, and converted to embedding vector through embedding layer (dim = 200). Then embedding vector passed to three layers of LSTM neural networks (dim = 512) and two linear layers (dim = 200), the output of which were latent vectors (dim = 200).

(2) $$\begin{array}{c} X_{e}=Embedding\left(X\right)\\ \left(h\_state_{1},c\_state_{1}\right)=Lstm_{1}\left(X_{e}\right)\\ \left(h\_state_{i},c\_state_{i}\right)=Lstm_{i}\left(h\_state_{i-1},c\_state_{i-1}\right)\\ \mu =Linear_{\mu }\left(h_{3}\right)\\ \log\;\sigma =Linear_{\log\;\sigma }\left(h_{3}\right)\\ z=\mu +e^{\log\;\sigma }\times \varepsilon \end{array}$$

In order to capture the correlation between molecule features and condition property, this paper proposes an FPC network based on attention mechanism (dim = 200,64,64), which can be categorized into three steps as follows:

(3) $$\begin{array}{c} Alignment:E_{\mathrm{zC}}=W\times \left[z;C\right]\\ Correlation\;Score:S_{\mathrm{zC}}=soft\max\left(E_{\mathrm{zC}}\right)=\frac{\exp\left(E_{z_{i}C}\right)}{\sum_{j=1}^{n}\exp\left(E_{z_{j}C}\right)}\left(n=N\left(z\right)\right)\\ Feature\;based\;correlation\;score:Z_{c}=S_{\mathrm{zC}}\times z \end{array}$$

Where *z* is the latent vectors, *C* is the corresponding condition property (QED in this study). [*z*; *C*]concatenated the molecule latent vectors with condition property, *W*is transformational weight matrix, *E*_zC_is the output of feature property correlation alignment step. The result of *S*_zC_ is obtained by further normalized *E*_zC_ with the softmax function, $S_{z_{i}C}$represents the correlation between molecule feature*z_i_* and condition property*C*, the larger of the value of $S_{z_{i}C}$ is, the stronger of their correlation is obtained, and vice versa. The latent vectors with correlation *Z*_c_ are obtained by multiplying the latent vectors *z*with the corresponding correlation score coefficient*S*_zC_.

The function of Decoder network was converted the latent vectors with condition property into SMILES with chemically valid and drug‐like property. This network was similar to the Encoder network (dim = 200, 512, 512, 512, the size of alphabet size, the size of alphabet size), except for the last softmax layer, which can be obtained characters in every position from the probability distribution.

(4) $$X_{c}=\mathrm{soft}\max\left(\mathrm{Line}\mathrm{ar}\left(\mathrm{Lst}m_{3}\left(\mathrm{Embe}\mathrm{dding}\left(Z_{c},C,X\right)\right)\right)\right)$$

The total loss function of the VAE_FPC molecule generation model is:

(5)

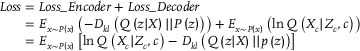




The VAE_FPC molecule generation model was trained until the early stop condition of molecule generation model, using a mini‐batch size of 64, utilizing the Adam optimizer, where the learning rate with 0.03 decay rate every 1000 steps, with the settings (α_0_ = 0.0005, β_0_ = 0.9, β_1_ = 0.999, ε = 10^−8^).

### Activity Prediction Model

For molecule fingerprints. Rdkit topological fingerprint (Rdkit) calculate the molecule subgraphs between minPath and maxPath, and it contain three kinds of information that includes atom types, aromaticity and bond types, which were encoding as numeric identifiers using a hash function.^[^ ^34^, ^35^ ^]^ With settings NumBits = 1024, MinPathLength = 1, MaxPathLength = 7, UseChirality = False, RootedFingerprint = False. Extended Connectivity Fingerprints with radius = 2 (ECFP4) was formed by setting a radius from a particular atom and counting the structure of neighborhoods to represent the molecular structure,^[^ ^36^, ^37^ ^]^ which contain information that include absolute charge, the number of heavy atomic connections and non‐hydrogen bond, atomic charge. With settings NumBits = 1024; Radius = 2; UseChirality = False. Avalon Fingerprint (Avalon) enumerates atom, bond, ring and feature pairs of the molecule subgraph and certain paths by using a molecule generator. All molecules were coding to fingerprint bit implicitly by using a hash function when they are enumerated. The detailed description of the feature categories was previously reported.^[^ ^38^ ^]^ The above‐mentioned three molecule fingerprints were calculated by rdkit.

### Machine Learning Models

*Support Vector Machine (SVM)*: The basic theory of which is found a hyperplane in a multidimensional space that maximizes margin between the support vectors every category, the support vectors are training samples on two category margins.^[^ ^39^, ^40^ ^]^ The main hyper‐parameters in that include kernel function, the regularization term C, the bandwidth parameter Gamma and whether to use heuristic contraction (shrinking). The optimal value of C ranges in from distribution of 0.001 to 1000 was optimized, kernel function that contains [linear, rbf, sigmoid, polynomial], the optimize value of Gamma ranges in from distribution 0.0001 to 8 or 1/features (the number of features). Random forest (RF). It is an ensemble algorithm that integrates multiple decision trees based on bagging, which aims to improve the generalization ability of the model by reducing the variance of individual decision trees.^[^ ^41^, ^42^ ^]^ For classification task, the final result in which the decision trees the most votes. For regression task, the final result can be obtained by the average of decision trees outputs.^[^ ^43^ ^]^ Three hyperparameters were optimized including the number of decision trees and maximum features and the maximum depth of the decision tree, which were optimized with values, [5, 20], [50, 100], [5, 30] the other parameters are default settings. Gradient Boosting Decision Tree (GBDT). It integrates multiple cart regression decision trees based on boosting, which combined the addition model with forward distribution algorithm.^[^ ^44^ ^]^ The decision tree was taken as the base function, which was constructed and finally integrated into a strong classifier after multiple iterations. Three hyperparameters were optimized including the number of decision trees, the maximum depth of the decision tree, the size of learning rate and subsample, which were optimized with values [50, 100], [5, 20], [0.05, 0.15], [0.7, 1.0], the other parameters are default settings.

The above three modeling methods were built in scikit‐learn and the optimal hyper‐parameters were gained from Tree Parzen Estimator (TPE), which converted the configuration space described by uniform, loguniform, quniform, and categorical variables into a nonparametric density distribution. Afterward, the different densities over the configuration space and different non‐parametric densities for observations were generated. The optimization objective was the highest of the average accuracy on training set and test set. Datasets were randomly divided into five parts for fivefold cross validation. The prediction results were averaged as the final results so as to improve the generalization ability and stability for prediction model.

*Feature selecting Methods*; Mutual information (MI) is a classical method commonly used to evaluate the correlation between feature variable and category variable. If the value of*MI*(*z_i_*; *y_i_*)is large, the correlation between the feature*z_i_* and the category*y_i_* is greater and vice versa. The relationship of which can be defined as:

(6) $$MI\left(Z;Y\right)=\sum_{z\in Z}\sum_{y\in Y}p\left(z,y\right)\log\frac{p\left(z,y\right)}{p\left(z\right)p\left(y\right)}$$

of which, *Z* ∈ {0, 1}, *Y* ∈ {0, 1}were represented the value of feature variable and category variable, the detailed description can be found in previous literature.^[^ ^45^ ^]^

Least absolute shrinkage and selection operator (Lasso) is a linear regression analysis method that carries out feature selection and regularization simultaneously. The core idea is to minimize the objective function, compress the coefficients with low correlation to zero, and then delete these characteristic variables so as to reduce the spatial dimension under the condition that the sum of the absolute value of regression coefficients is less than a threshold.^[^ ^46^, ^47^ ^]^ The alphas (regularization coefficient) was optimized from 0.001 to 0.1 with step 0.001 and the other parameters are default settings.

### Model Performance Evaluation

*Classification task*: Prediction Accuracy (Acc), F1‐Score were applied to evaluate the classification model performance:

(7) $$Acc=\frac{TP+TN}{TP+FP+TN+FN}$$

(8) $$F_{1}-score=2\times \frac{precision\times recall}{precision+recall}=\frac{2TP}{2TP+FN+FP}$$

*Acc* ∈ [0, 1]reflects the ratio of accurate prediction samples, *F*_1_ − *score* ∈ [0, 1] is the harmonic mean of recall and precision. The bigger the above two metrics are, the better the model performance will be.

*Regression task: MRE*, *MAE*, *RMSE*were applied to evaluate regression model performance. The smaller the followings three metrics are, the better the model performance will be.

(9) $$MRE=\frac{1}{N}\sum_{i=1}^{N}\left|\frac{{\hat{y}}_{i}-y_{i}}{y_{i}}\right|$$

(10) $$MAE=\frac{1}{N}\sum_{i=1}^{N}\left|{\hat{y}}_{i}-y_{i}\right|$$

(11) $$RMSE=\sqrt{\frac{1}{N}\sum_{i=1}^{N}{\left({\hat{y}}_{i}-y_{i}\right)}^{2}}$$

*Chemical Space and similarity Analysis*: To visually generate chemical spatial maps of molecules and CRC dataset or AD dataset, t‐Distributed Stochastic Neighbor Embedding (T‐SNE) technique was implemented. The fundamental idea of which is to reduce the high dimensionality into the low‐dimensional feature space by using nonlinear map: i) Morgan fingerprint (with settings: radius = 2, nBits = 2048) was calculated by rdkit for representation each molecule; ii) T‐SNE technology to reduce the dimensions of the Morgan fingerprint from 2048 to the 2 dimensions. T‐SNE used by scikit‐learn with the fault settings. Tanimoto similarity on Morgan fingerprint (with settings: radius = 2, nBits = 2048) was applied to quantify the structural similarity between generated novel molecules and the known active molecules, which ranges from 0 to 1, the higher the value, the more structure similar it is.^[^ ^8^, ^10^ ^]^ SciFinder (version2021, Chemical Abstracts Service, https://scifinder.cas.org) was filtered the structural novelty for de novo design molecules.

### Molecule Generation Model Evaluation

(12) $$Validity=\frac{N_{\mathrm{Valid}}}{N_{\mathrm{Sample}}}\times 100\%$$

(13) $$Uniqueness=\frac{N_{\mathrm{Unique}\_\mathrm{Valid}}}{N_{\mathrm{Valid}}}\times 100\%$$

(14) $$QED_{\mathrm{request}}=\frac{N_{\mathrm{Unique}\_\mathrm{Valid}\_\mathrm{QED}>0.6}}{N_{\mathrm{Unique}\_\mathrm{Valid}}}\times 100\%$$

(15) $$Num_{\mathrm{QEDrequest}}=N_{\mathrm{Sample}}\times Validity\times Uniqueness\times QED_{\mathrm{request}}$$

(16) $$Lead_{\mathrm{request}}=\frac{N_{\mathrm{Unique}\_\mathrm{Valid}\_\mathrm{QED}>0.6\_\mathrm{Activity}}}{N_{\mathrm{Unique}\_\mathrm{Valid}\_\mathrm{QED}>0.6}}\times 100\%$$

(17) $$Num_{\mathrm{Lead}}=N_{\mathrm{Sample}}\times Validity\times Uniqueness\times QED_{\mathrm{request}}\times Lead_{\mathrm{request}}$$

(18) $$Novelty=\frac{N_{\mathrm{Unique}\_\mathrm{Valid}\_\mathrm{QED}>0.6\_\mathrm{Activity}\_\mathrm{not}\;\mathrm{in}\;\mathrm{trainingset}}}{N_{\mathrm{Unique}\_\mathrm{Valid}\_\mathrm{QED}>0.6\_\mathrm{Activity}}}\times 100\%$$

(19) $$\begin{array}{ccc} Num_{\mathrm{Novel}\_\mathrm{Lead}} & = & N_{\mathrm{Sample}}\times Validity\times Uniqueness\times QED_{\mathrm{request}}\\ & & \times Lead_{\mathrm{request}}\times Novelty \end{array}$$

*N*_Sample_ = 64in this paper, the results were averaged for ten repetitions when batch size molecules were sampled at each repetition. Validity represents the percentage of generated molecules that satisfies chemically valid, such as smiles internal syntax rule (opening and closing branches or rings, allowed valence, etc). Uniqueness is the fraction of the valid molecule that does not duplicate. $N_{\mathrm{Unique}\_\mathrm{Valid}\_\mathrm{QED}>0.6}$,$N_{\mathrm{Unique}\_\mathrm{Valid}\_\mathrm{QED}>0.6\_\mathrm{Activity}}$are the amount of sample molecules that satisfies chemically valid and drug‐like property (QED>0.6) and activity at the same time, respectively. *Num*_Lead_is the amount of lead compound. Novelty is the proportion of novel molecules and not in the training and finetune sets, $Num_{\mathrm{Novel}\_\mathrm{Lead}}$is the amount of novel structured lead compound.

### Early Stop

Early stop was used to reduce training time consumption and avoid overfitting. In the VAE_FPC molecule generation model training process, maximum epoch was set to 8 epochs, or if the accuracy of 500 consecutive steps is higher than the threshold δ (δ = 0.95in this paper), the training procedure was terminated early. However, this early termination criteria was empirical and could be changed based on the learn tasks. In the transfer learning process, if the average loss of every epoch is no longer decreasing for continuous N epoch, the model will stop training and the optimal model parameters with the minimum average loss will be retained, the parameter N of early stop in transfer learning training process of different target domains were obtained through experimental study (Figures S3 and S4, Supporting Information). The early stop condition of PRTL model is that the maximum number of recurrences is 50 or no novel molecules meet the desired properties were generated, that is the target domain does not update.

### Partition Recurrent Transfer Learning (PRTL) Model

With the emergence of more machine learning application scenarios, the existing well‐performing supervised learning needs a large amount of annotated data, which is a tedious and costly task. Therefore, much has been paid to transfer learning because it can be trained with small data sets and learning to bias target property. The goal of transfer learning is to learn general features on the large dataset, which also be useful for the second task in the smaller data set. In order to improve the efficiency of generating active molecules, this paper proposes partition transfer learning (PTL) method (Figure S1, Supporting Information). Finetune dataset can be classified by taking QED, IC_50_ as drug‐like and activity evaluation index, respectively. The four sub‐datasets were respectively used as target domains for transfer learning, PTL with high activity sub‐partition target domain was taken as an example: first, C dataset was used as the target domain, transfer learning was trained based on VAE_FPC model parameters until the early stop condition of PTL was reached. Then, on the basis of this model, PTL was carried out with A dataset as the target domain, the sampling procedure was performed after that. The similar procedure for PTL low activity sub‐partition target domain, the compared experiments with the overall dataset as target domain as shown in Tables S5–S7 (Supporting Information) for CRC target domain and Tables S19–S21 (Supporting Information) for AD target domain. This indicates that in the transfer learning stage, the more focus on the molecule properties of the target domain are, the better transfer learning effect will be, but the number of molecules should not be too small. To improve the novelty of the generated molecules, this paper proposes the PRTL method that added two updates strategies to PTL method, which is model parameters update on the training transfer learning model and target domain dataset update (Figure S2, Supporting Information). PRTL with high activity sub‐partition target domain can be taken as an example. The sampling program was implemented when the early stop condition of PTL was reached, batch size molecules were sampled at each repetition and continue ten repetitions. Then the repeated molecules and the existing molecules of the training dataset and finetune dataset were removed and the remaining molecules were used to update the target domain, meanwhile, the model parameters at the end of the training were updated. The whole process was repeated until the condition of PRTL early stop was met. The experimental results are shown in Table S8 (Supporting Information) for CRC target domain and Table S22 (Supporting Information) for AD target domain.

### Information of Compounds

Novel compounds (4‐(3‐ethoxy‐4,5‐dimethoxyphenyl) −3‐(p‐tolyl)−1H‐ pyrazole (compound 1901), 1‐(3‐chloro‐4‐ethoxyphenyl)−5‐(3,4,5‐ trimethoxyphenyl)−1H‐ tetrazole (compound 2238), 3,5‐diethyl‐4‐(4‐hydroxy phenethyl) phenol (compound 548), 3‐(4‐hydroxyphenethyl)−4‐propylphenol (compound 398)) were synthesized, and the details are presented in Supporting Information.

1,2,3,4‐Tetrahydrocyclopent[b]indol‐2‐amine (compound 600, CAS 1263284‐26‐9, catalog no. T847650), 3‐Aminothieno[2,3‐b]pyridine‐2‐ carboxylic acid ethyl ester (compound 3141, CAS 52505‐46‐1, catalog no. E857975), and 2‐Phenyl‐4(3H)‐quinazolinone (compound 2524, CAS 1022‐45‐3, catalog no. T17142) were purchased from Shanghai yuanye Bio‐Technology Co., Ltd. 5‐(4‐hydroxyphenethyl)benzene −1,3‐diol (compound, 574, CAS 58436‐28‐5, catalog no. wkq‐02349), [1,1′‐biphenyl]−4‐ol (compound 467, CAS 92‐69‐3, catalog no. wkq‐00737), 7,8‐dimethoxy‐2H‐chromen‐2‐one (compound 698, CAS 2445‐80‐9, catalog no. wkq‐03396) and 4‐allyl‐1,2‐dimethoxybenzene (compound 571, CAS 93‐15‐2, catalog no. wkq‐02191) were purchased from Sichuan Victory Biological Technology Co., Ltd.

### Administration of Compounds

For cell experiment, the compound was dissolved in dimethyl sulfoxide (DMSO) (Sigma, D5879) as a reserve solution (100 mm) and the reserve solution was diluted to the indicated concentrations using DMED. For in vivo anti‐colorectal cancer efficacy experiment study, compounds were suspended in saline (Sigma, S0817). For in vivo anti‐inflammation and anti‐AD efficacy experiments, compounds were suspended in 0.5% (w/v) carboxymethylcellulose sodium (Sigma, C5678).

### Animal Management

BALB/c nude mice were used for in vivo anti‐cancer efficacy experiment and purchased from Beijing HFK bioscience co., Ltd. C57BL/6 mice were used for in vivo anti‐inflammation and anti‐AD efficacy experiment and purchased from Liaoning Changsheng biotechnology co., Ltd. These mice had free access to eat and drink under the standard condition (a 12 h light/12 h dark cycle). The experiment was carried out under the approval of Animal and Medical Ethics Committee of Northeastern University. For in vivo anti‐cancer efficacy experiment, the approval number is NEU‐EC‐2022A032S. For in vivo anti‐inflammation efficacy experiment, the approval number is NEU‐EC‐2021A025S. For in vivo anti‐AD efficacy experiment, the approval number is NEU‐EC‐2021A022S.

*In Vivo Anti‐Colorectal Cancer Efficacy*: The colorectal cancer‐bearing animal model was established. The experimental design is shown in Figure 3A. HT29 cells were injected into the right flank of BALB/c nude mice. The tumor size was measured once every two days using a caliper (tumor volume = 1/2 × shortest diameter^2^ × longest diameter). 1901 (1, 2.5, and 5 mg kg^−1^) was given by intravenous injection every other day. The body weight was also recorded once every two days. After treatment, animals were anesthetized, and the tumor and organs for liver and spleen were obtained. The tumor weight, tumor burden and tumor inhibition rates of animals were calculated. The tumor was also performed HE staining. The spleen index was calculated by the following formula: weight of spleen/weight of body × 100%. Commercial kits were used to test ALT level (Nanjing Jiancheng, C010‐2‐1) and AST level (Nanjing Jiancheng, C009‐2‐1) for liver according to the manufacturer's instructions.

*In Vivo Anti‐Inflammation Efficacy*: Animals were anesthetized with 2.5% (w/v) tribromoethanol (1 mL 100 g^−1^ body weight) via intraperitoneal injection. LPS was dissolved in saline (40 µg mouse^−1^, 2 µL) and performed intrahippocampal injection at specified location relative to the bregma (AP: −2.4 mm, ML: ±2.0 mm, DV: −2.2 mm). Animals were grouped randomly. Mice in control group underwent saline‐injection plus oral vehicle treatment, mice in LPS group underwent LPS injection plus oral vehicle treatment, mice in 548 treatment groups underwent LPS injection plus oral 548 (5, 10, and 20 mg kg^−1^) treatment.

*In Vivo Anti‐AD Efficacy*: Animals were anesthetized as described above. Aβ_1‐42_ peptide was prepared to obtain Aβ_1‐42_ oligomers, dissolved in PBS, and performed lateral ventricle injection at specified location relative to the bregma (AP: −0.5 mm, ML: −1.1 mm, DV: −2.5 mm). Aβ_1‐42_ oligomers (10 pmol) were injected (1 µL min^−1^) and the syringe was kept for 5 min to allow diffusion. Animals were randomly divided into five groups. Mice in control group underwent PBS‐injection plus oral vehicle treatment, mice in Aβ_1‐42_ group underwent Aβ_1‐42_ oligomers injection plus oral vehicle treatment, mice in 548 treatment groups underwent Aβ_1‐42_ oligomers injection plus oral 548 (1 mg kg^−1^, 2.5 g kg^−1^, and 5 mg kg^−1^) treatment.

### Behavioral Assessment

For anti‐AD efficacy 0model, behavioral assessments were performed and the experiment schedule are shown in Figure 6A.

Y‐Maze test was conducted on the thirteenth day. Spontaneous alternation is defined as the consecutive entries into the three arms in the Y‐maze test. The dimensions of each arm of the maze were 38 cm in length, 12 cm in height, and 5 cm in width, all converging at an equal angle. During an 8 min session, each mouse was placed at the end of one arm and allowed unrestricted movement throughout the maze. The total number of arm entries and the occurrence of alternation were recorded. The cumulative count of arm entries was collected over a duration of 8 min. The percentage of alternation was calculated.

Novel object recognition task was conducted from the fourteenth to the eighteen day. The experimental setup consisted of a plastic arena in an open field format, with dimensions of 44 × 44 × 44 cm. The experiment was comprised of three distinct phases: habituation, acquisition, and test. During the habituation phase, the mice were introduced to the arena and allowed to explore it for 3 min, twice a day, to become familiar with the environment. Following the habituation phase, the acquisition phase began, in which the mice had the opportunity to examine two identical objects for a duration of 5 min. The total time spent exploring both objects was recorded. To calculate the recognition index as the following formula: time spent exploring one of the identical objects/total time spent exploring both objects × 100%. Moving on to the test phase, 1 h after exploring the identical objects, the same mouse underwent a 5 min trial. However, this time a different object was substituted for one of the initially identical objects. The time spent exploring both objects was recorded. The discrimination index was calculated using the formula: [(Tn−Ti)/(Tn + Ti)] × 100%. The time spent in detecting the new object is denoted as Tn, while time spent in detecting one of the identical objects is denoted as Ti.

Morris water maze test was conducted from the nineteenth to the twenty‐fifth day. Morris water maze comprised of a circular pool divided into four quadrants. Positioned in the center of one specific quadrant was a transparent cylindrical platform, located below the water surface. The Morris water maze test encompassed two assessments: the place navigation test and the probe test. During the place navigation test, the mice were subjected to trials each day. At the beginning of every trial, the mice were placed in the water, facing the wall of the pool, from one of four distinct starting points. Subsequently, they were given the freedom to swim and search for the hidden platform within the pool. If a mouse successfully discovered the platform within 120 s, it remained on the platform for an additional duration of 10 s. However, if a mouse failed to locate the platform within the specified time, it was gently positioned onto the platform for a duration of 10 s. In order to ascertain the escape latency, the time taken by each mouse to reach the platform and remain on it for at least 3 s was measured. Each trial concluded once the mouse reached the platform or after the elapse of 120 s. The escape latency was calculated and recorded for each mouse. Following the completion of the place navigation test, a single probe test was conducted on the subsequent day. During this trial, the platform was removed from the pool. The mice were granted a time limit of 60 s to navigate the maze, commencing from a position opposite to the original location of the platform. The related parameters were recorded.

### Cell Culture

HT29 (human colon carcinoma cell line) and BV‐2 (rodent microglial cell line) cells were used for in vitro experiments. Culture condition was under a humidified incubator of 37 °C with 5% CO_2_. The culture medium was DMEM (Gibco, C11965500BT) supplemented with 10% FBS (Procell, 16421–50), 100 U mL^−1^ penicillin and 100 µg mL^−1^ streptomycin (Promega, 03‐031‐1B).

### Cell Viability Assay and Nitrite Assay

For cell viability assay, following experimental procedures, the cell culture medium was removed, and subsequently the cells were subjected to incubation with a solution containing 3‐[4,5‐dimethylthiazol‐2‐yl]‐ 2,5‐diphenyltetrazolium bromide (MTT) at a concentration of 0.2 mg mL^−1^ for a duration of 3 h at a temperature of 37 °C. To solubilize the formazan crystals formed within the cells, DMSO was added. The absorbance was measured at the wavelength of 490 nm. NO synthase activity was determined by measuring the accumulation of nitrite. For in vivo nitrite assay, brain tissues were homogenized and the nitrite content was measured by a NO assay kit (Nanjing Jiancheng, A013‐2‐1) according to the instructions in the manual. For in vitro nitrite assay, Griess reagent (Beyotime, S0021S) was used. At room temperature, a combination of 50 µL culture supernatant and 50 µL Griess reagent was mixed. The measurement was performed at a wavelength of 540 nm with an absorbance microplate reader.

### Immunofluorescence and Western Blot

The fixation of brain tissue samples was performed using a 4% paraformaldehyde solution. Then the tissues were dehydrated with 20% and 30% sucrose and cut into 10 µm thick slices. The slices were blocked with 5% goat serum for a duration of 2 h. Antibody against Iba‐1 (1:500; Abcam, ab283319) was applied to incubate brain slices overnight, following a fluorescein isothiocyanate (FITC)‐labeled secondary antibody was utilized to incubate brain slices for 2 h. The resulting fluorescent images were captured using Leica DMI3000B fluorescence microscopy. For western blot, RIPA lysis buffer (Beyotime, P0013B) containing protease inhibitor cocktail (Bimake, B15001) was utilized to extract total proteins from both tissues and cells. The protein concentration in each sample was determined using the BCA protein assay kit (Beyotime, P0011). Subsequently, the proteins were separated using SDS‐PAGE gels and transferred onto PVDF membranes (Immobilon‐P, IPVH00010). Anti‐GSS (abcam, ab133592), GPX4 (abcam, ab125066), iNOS (abcam, ab3523) or β‐actin (abcam, ab8227) was used to incubate the membranes at 4 °C overnight, then incubated with peroxidase‐conjugated secondary antibody for 1 h at room temperature. Protein levels were normalized to β‐actin. Image Lab software was used to acquire and analyze blot images.

### CETSA and DARTS

For CETSA,^[^ ^48^ ^]^ cells were collected into a centrifugal tube and subjected to freeze‐thawing. The cell lysates were then incubated with specific compounds or an equal volume of DMSO at 25 °C for 1.5 h. Subsequently, they were individually heated at indicated temperatures (ranging from 42 to 70 °C). The cell lysates were centrifuged at 20 000 g for 20 min at 4 °C, and the soluble fractions were isolated for SDS‐PAGE. Protein bands showing significant differences after treatment with the compounds were selected and subjected to trypsin digestion. The trypsin‐digested peptides were then analyzed using mass spectrometry. For DARTS,^[^ ^48^ ^]^ cells were lysed in M‐per lysis buffer (Thermo, 78 501) containing phosphatase and protease inhibitor (Bimake, B15001) and divided into aliquots. After centrifugation, the supernatant was collected and TNC butter was added. The mixture was then incubated with DMSO or compound for an additional 1 h. Pronase was added at a ratio of 1:1000 for 30 min, and the reaction was terminated by adding protease inhibitor. Finally, the samples were analyzed by Western blot.

### Molecular Docking

Glide tools in Maestro‐2018 were used for the molecular docking. The iNOS protein structure (PDB ID: 4NOS) was taken from the Protein Date Bank. The structure of iNOS was loaded into Maestro‐2018 and then the structure was optimized by Protein Preparation Wizard Tab. Briefly, water molecules were removed from the protein structure and hydrogen atoms were added. In this crystal structure, iNOS was co‐crystallized with methyl (3S)−3‐{2‐[(1,3‐benzodioxol‐5‐ylmethyl) amino] −2‐oxoethyl}−4‐[2‐ (1H‐imidazol‐1‐yl) pyrimidin‐ 4‐yl]piperazine‐1‐carboxylate, and the binding sits was chose as active sits. Then compounds were loaded into Maestro‐2018, respectively. LigPrep Panel was used to optimize ligands. For precision of molecular docking, XP was selected for docking. After docking process, the score was recorded and further analysis was conducted.

### Iron Content, GSH Level Measurements, and ROS Production

The level of free iron was measured using Iron Assay Kit (Nanjing Jiancheng, A039‐2‐1). GSH levels were analyzed using GSH assay kit (Beyotime, S0052). The production of ROS in cells was tested using Dihydroethidium (Beyotime, S0063). The tissue and cells were prepared according to the manufacturer's instructions, respectively.

### Lipid Peroxidation Assay

BODIPY 581/591 C11 (Invitrogen, D3861) was used to observe the levels of lipid peroxides. Briefly, cells were incubated with BODIPY 581/591 C11 (10 µm) for 30 min behind the scenes and then washed with PBS. The lipid ROS was measured according to the manufacturer's instruction. For MDA measurement, the tissue and cells were prepared according to the manufacturer's instructions, MDA assay kit (Beyotime, S0131M) was used to exam the lipid peroxidation products malondialdehyde level.

### Statistical Analysis

Data were expressed as mean±SEM and analyze using SPSS 22.0 software. For comparison between two groups, the *t*‐test was used. One‐way ANOVA followed by Bonferroni's *post hoc* test was used to analyze the dosage effect of compound among three or more groups. Two‐way ANOVA followed by Bonferroni's *post hoc* test was used to analyze multi‐factors comparisons of the parameters. *P* < 0.05 was regarded as statistically significant.

## Conflict of Interest

The authors declare no conflict of interest.

## Author Contributions

D.H. and Q.L. contributed equally to this work. D.H. and Y.H. led the project, designed and planned the experiments. Q.L., C.H., and J.W. developed and implemented the DTLS. Y.M., Q.M., L.X., N.L., Y.Y., J.L. and L.W. performed in vivo and in vitro experiments, and analyzed the data. Y.L. and H.C. conducted novel structured compounds synthesis. Q.L. and Y.M. wrote the manuscript. Q.M. and Y.M. performed data collection and managed the literature retrieval.

## Supporting information

Supporting Information

Supporting Information

Supporting Information

## Data Availability

The data that support the findings of this study are available from the corresponding author upon reasonable request.
