# Supplementary material for: De Novo Generation and Identification of Novel Compounds with Drug Efficacy Based on Machine Learning
Source: Adv Sci (Weinh). 2024 Jan 10;11(11):2307245. doi: 10.1002/advs.202307245 (PMC10962488; doi:10.1002/advs.202307245)
Supplement: Supplementary file 1 — Supporting Information [file ADVS-11-2307245-s003.pdf]

ADVANCED SCIENCE
Open Access

Supporting Information

for *Adv. Sci.*, DOI 10.1002/advs.202307245

De Novo Generation and Identification of Novel Compounds with Drug Efficacy Based on Machine Learning

Dakuo He, Qing Liu, Yan Mi, Qingqi Meng, Libin Xu, Chunyu Hou, Jinpeng Wang, Ning Li, Yang Liu, Huifang Chai, Yanqiu Yang, Jingyu Liu, Lihui Wang and Yue Hou**

Supplementary Figures

Legend

Figure S1. The overall framework of PTL. VAE_FPC model was retrained on finetune dataset that can be classified by taking QED, IC_{50} as drug-like and activity evaluation index, respectively.

Figure S2. The overall framework of PRTL. PRTL adds model parameters update on the training transfer learning model and target domain dataset update to PTL method to improve the novelty of the generated molecules.

Figure S3. The comparison diagram of early stop with different patience for CRC target domain. The optimal early stop value of PTL was Nbest =2 for all subsets, and as this parameter increased, overfitting occurred.

Figure S4. The comparison diagram of early stop with different patience for AD target domain. The optimal early stop value of PTL was Nbest =2 for ABCD, Nbest =3 for One_AB, Two_AB, Two_A, Two_B subsets, and Nbest =5 for One_A, One_B subsets.

Figure S5. The similarity distribution between generated novel compounds with those molecules in CRC/AD target domain, respectively. A-B: Tanimoto similarity

between 1901 and 2238 with every molecule in CRC target domain. **C-D:** Tanimoto similarity between 548 and 398 with every molecule in AD target domain.

Figure S6. Effect of 1901 on ferroptosis and 1901 target analysis. A: KEGG enrichment analysis of differently expressed genes in 1901-treated HT29 cells. B: The effect of compound 1901 on cell viability was measured combined with apoptosis inhibitor Z-VAD-fmk (10 μM, 72 h), autophagy inhibitor 3-MA (1 mM, 72 h) or pyroptosis inhibitor disulfiram (30 μM, 72 h). N=3 independent cell batches. $^{*}P < 0.05$, $^{***}P < 0.001$ compared with the indicated group. **C:** The potential target protein of compound 1901 was identified as glutathione synthetase (GSS) using LC-MS/MS analysis. **D:** Binding mode and site between compound 1901 and GSS were shown by molecular docking analysis.

Figure S1

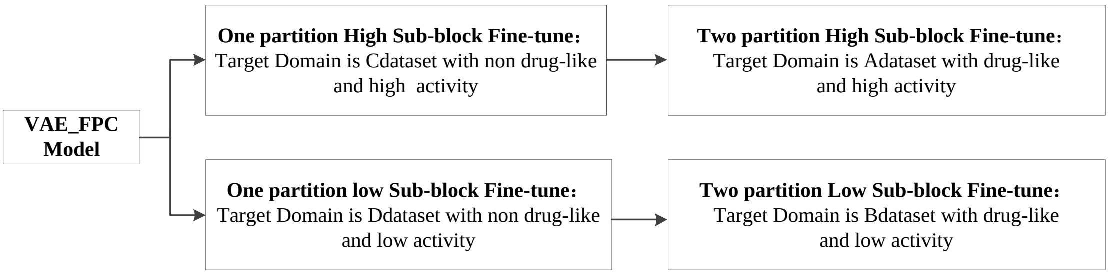

Figure S2

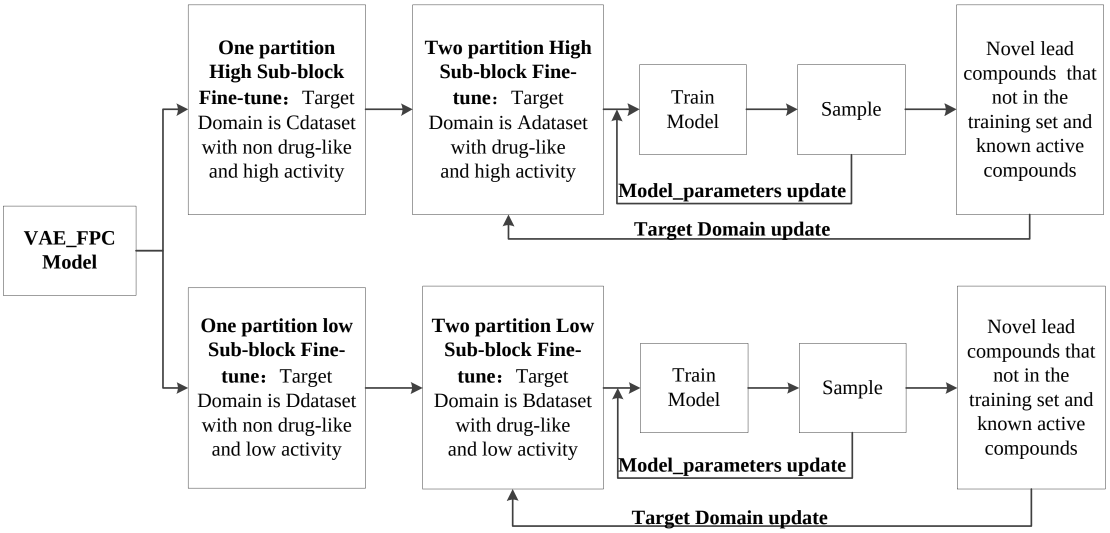

Figure S3

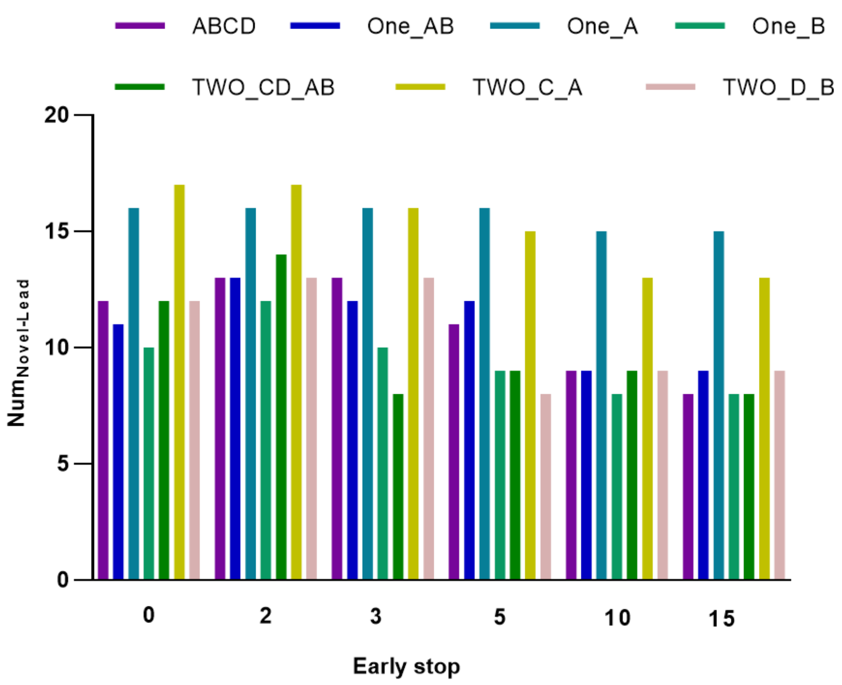

Figure S4

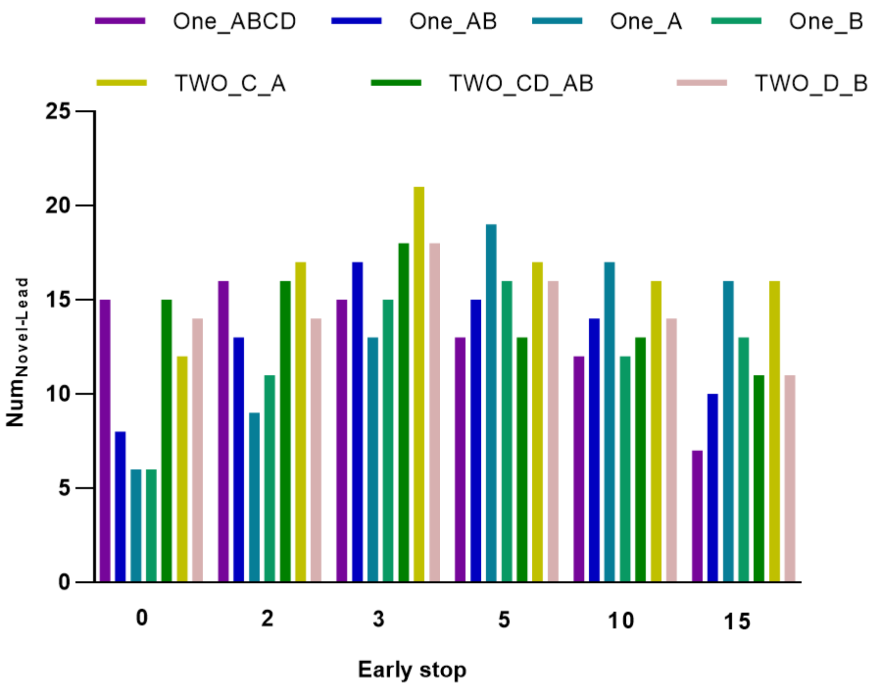

Figure S5

A

B

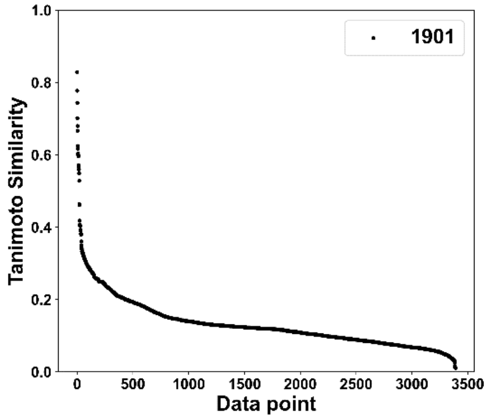

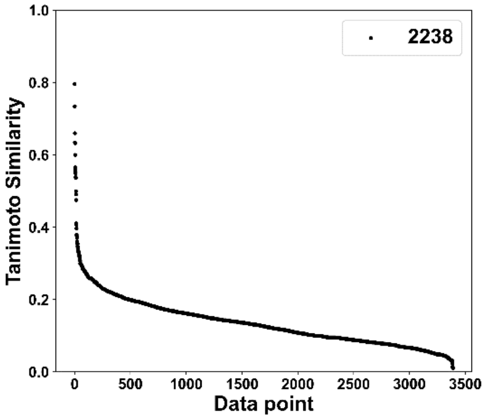

C

D

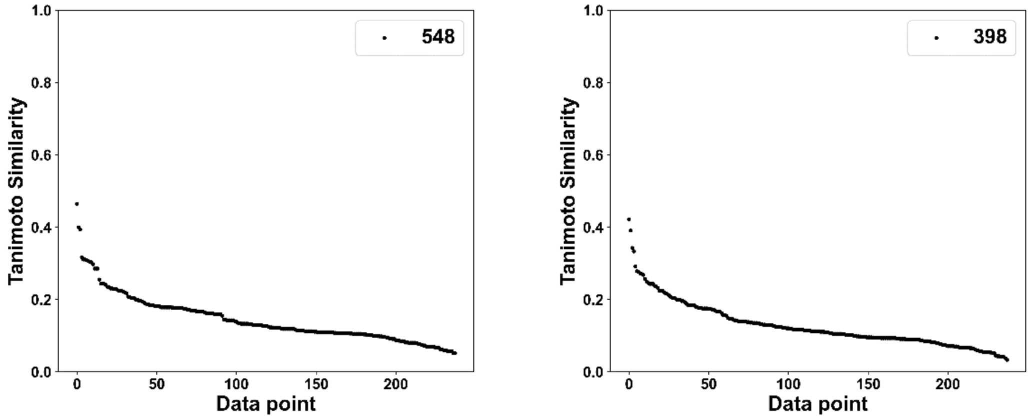
1.0
0.8
0.6
0.4
0.2
0.0
Tanimoto Similarity
548
0
50
100
150
200
Data point
398
Data point

Figure S6

A

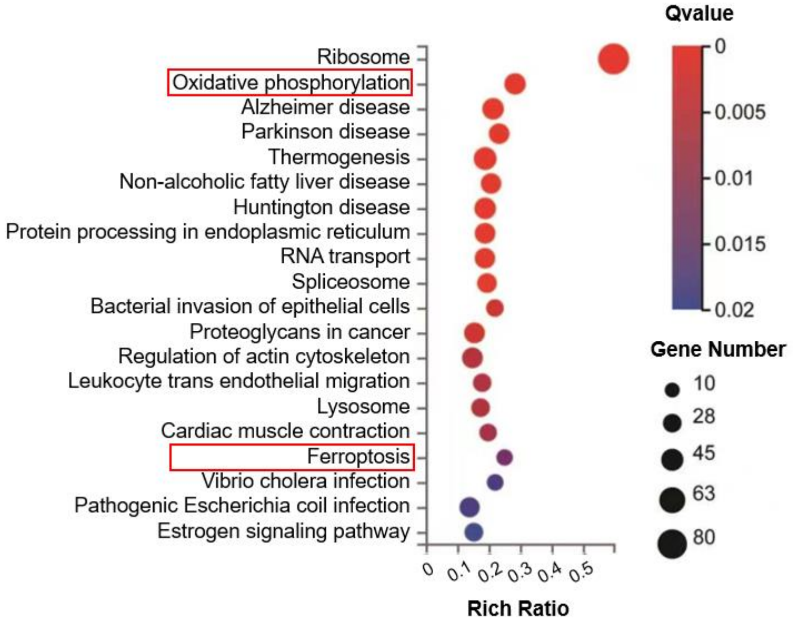
Ribosome
Oxidative phosphorylation
Alzheimer disease
Parkinson disease
Thermogenesis
Non-alcoholic fatty liver disease
Huntington disease
Protein processing in endoplasmic reticulum
RNA transport
Spliceosome
Bacterial invasion of epithelial cells
Proteoglycans in cancer
Regulation of actin cytoskeleton
Leukocyte trans endothelial migration
Lysosome
Cardiac muscle contraction
Ferroptosis
Vibrio cholera infection
Pathogenic Escherichia coil infection
Estrogen signaling pathway
0
0.1
0.2
0.3
0.4
0.5
Rich Ratio
Qvalue
0
0.005
0.01
0.015
0.02
Gene Number
10
28
45
63
80

B

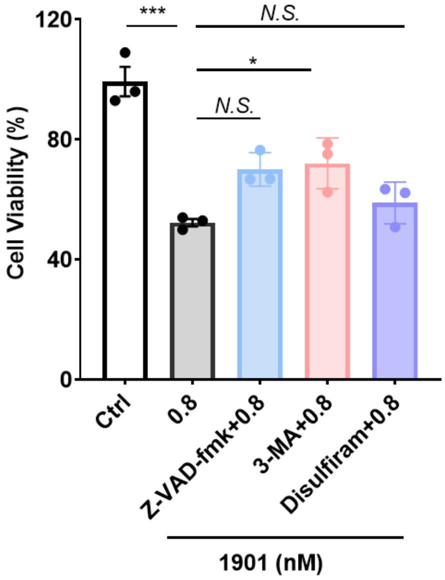

120
80
40
0
Cell Viability (%)
***
N.S.
*
N.S.
Ctrl
0.8
Z-VAD-fmk+0.8
3-MA+0.8
Disulfiram+0.8
1901 (nM)

C

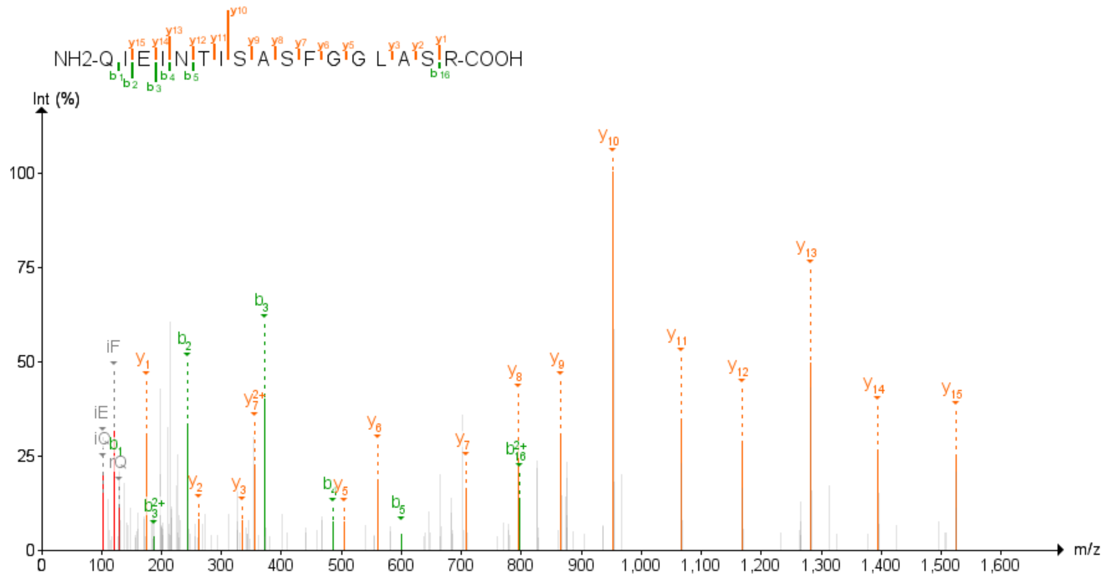

NH2-Q I E I N T I S A S F G G L A S R-COOH
Int (%)
m/z

D

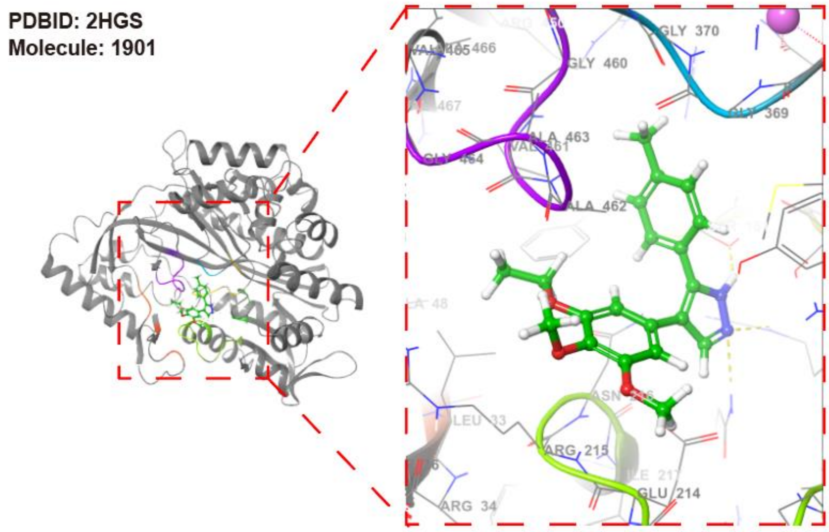

Supplementary Tables

Comparative experimental on generation models. The comparison of molecule generation approaches was important for the drug design domain based on deep learning, we compared the model performance of VAE_PFC network with variational auto-encoder (VAE) [1], conditional variational auto-encoder (CVAE) [4], recurrent neural network (RNN) [2], long short-term memory (LSTM) [3], gate recurrent unit (GRU). The comparison results for CRC molecule generation model were shown in the Table S1, compared with VAE molecule generation model, the Uniqueness indicator has reduced by 0.16%, the Validity indicator went up 0.62%, and improved by 44.19% for the QEDrequest. That's because FPC network was to learn the correlation between condition property and latent vectors. In order to conduct a fair comparative experiment, the dataset of QED higher than 0.6 in the training set were used to train the molecule generation model, which shown in Table S1, entry2-entry5. Compare with VAE_0.6, Uniqueness indicator has reduced by 0.16%, the Validity indicator went up 0.47%, and

improved by 3.16% for the QEDrequest, the validity of FPC network was further verified. Compare with RNN_0.6, LSTM_0.6, GRU_0.6, the Validity and $QED_{request}$ has improved to varying degrees. Among them, the Validity of RNN_0.6 was low. Compare with the CVAE, QED was used as the conditional property in this study. Compared VAE_FPC and CVAE (entry7), the Validity, Uniqueness, QEDrequest has improved 0.16%, 0.15%, 3.32%, respectively. The comparison results for AD molecule generation model were shown in the Table S12. Compared with VAE molecule generation model (entry1), the Validity indicator went up 0.40%, and improved by 49.48% for the QEDrequest. Compare with entry2-entry5, QEDrequest improved by 3.57%, 7.91%, 5.15%, 6.19%, respectively. Compare with the CVAE (entry7), the QEDrequest went up 4.42%, these experimental results further validated the effectiveness of the FPC network.

CRC activity classification model (CRCACM). The modeling data were derived from the CRC dataset, where the number of active and inactive molecules against CRC was 3390 and 2887, respectively. Three molecule fingerprints (Rdkit, ECFP4, Avalon) as input and corresponding output was activity label ($Y \in \{0,1\}$ if IC_{50}<10um, the corresponding label was 1, vice versa), the model performance of three modeling methods were compared as shown in Table S2-4. For SVM and RF, GBDT modeling methods, the molecule representation corresponding to the optimal model performance was Avalon fingerprint. Subsequently, compared the model performance of Avalon_SVM, Avalon_RF, Avalon_GBDT, among them, Avalon_GBDT has the best model performance for CRCACM. The accuracy, F1-score of test set was 84.79%,

0.858, the optimal model-parameters in Table S25.

CRC activity prediction model (CRCAPM). The modeling data were derived from the active molecules against CRC, filter and retain the data that has a specific pIC_{50} value (3359). Three molecule fingerprints (Rdkit, ECFP4, Avalon) as input and corresponding output was pIC_{50} value, the model performance of three modeling methods were compared as shown in Table S9-11. Among them, Avalon_GBDT performed best, the MRE, MAE, RMSE of training set was 1.30%, 0.08, 0.222, the MRE, MAE, RMSE of test set was 6.86%, 0.42, 0.644, respectively. The introduction of feature selection method cannot further improve the model performance for CRCACM and CRCAPM, the optimal model-parameters in Table S25.

AD activity classification model (ADACM). The modeling data were derived from the active molecules against AD and molecules that not active against any target were randomly selected from ChEMBL database. First, we evaluated the impact of three molecule fingerprints coupled with three modeling methods on the performance of ADACM. Table S13-15 compares of the model performance evaluation that were the accuracy, F1-score of training set and test set for three molecule fingerprints based on SVM, RF, GBDT modeling methods. Consider the average of accuracy, F1-score of the training sets and test sets, for SVM modeling method, the Rdkit fingerprint has better prediction performance, for RF and GBDT modeling method, the Avalon fingerprint has the better prediction performance. Subsequently, the performance of the three modeling methods (Rdkit_SVM, Avalon_RF, Avalon_GBDT) was compared, the experiment results showed that Avalon_RF was slightly superior to the other two

models.

Then we evaluated two different feature selection categories that MI, Lasso coupled with three modeling methods, which were shown in Table S16-18, it can be seen that the average value of classification model performance has improved, which verified the validity of feature selection for ADACM. Among which the Lasso feature selection method achieved the optimal prediction performance. In terms of the average of model performance of accuracy and F1-score, compared to Lasso_Rdkit_SVM, Lasso_Avalon_RF, Lasso_Avalon_GBDT, the prediction results of Lasso_Rdkit_SVM has optimal classification performance for the ADACM. The accuracy, F1-score of test set was 98.32%, 0.983, respectively. The optimal alpha was 0.005 and the other model parameters as shown in Table S26.

The comparison experiment of early stop. Evaluate the amount of generated novel molecules with desired properties as a function of the size of N (N=0,2,3,5,10,15 in this study), the experimental results were shown in the Figure S3 and Figure S4, the following conclusions can be drawn: (i) the optimal early stop value of PTL was N_{best} =2 for all subsets of the CRC target domain, and as this parameter increased, overfitting occurred. (ii) the optimal early stop value of PTL was N_{best} =2 for ABCD, N_{best} =3 for One_AB, Two_AB, Two_A, Two_B subsets, and N_{best} =5 for One_A, One_B subsets of the AD target domain.

The comparative experimental results by using PRTL with CRC target domain. Comparative results of that knowledge transfer from VAE_FPC molecule generation model to whole known actives (entry1)[3] and partition known actives (entry2) as shown

in Table S5, experimental results shown that except for Validity and Novelty, other indicators were improved, among them $QED_{request}$ and $Lead_{request}$ increased by 5.27% and 1.79% respectively, the reason for this was that the $Domain_{ABCD}$ was classified $Domain_{CD}$ and $Domain_{AB}$ by drug-like index, indicating that the more focused the target attribute of molecules in the target domain, the better the effect of transfer learning. Based on this concept, this study proposed PTL method.

As shown in Table S6, the comparative sampling results of the target domain was $Domain_{CD\_AB}$ (Table S5, entry2) and $Domain_{AB}$ (Table S6, entry1) after transfer learning, the generate novel molecules that satisfied the desired properties were improved. Except for the Uniqueness and QEDrequest, other indexes were improved, among them, the most improvement was $Lead_{request}$, which improved by 2.84%, this was because the $Domain_{CD}$ biologically activity knowledge can be learned, which verified the effectiveness of PTL model. Compared the sampling results of transfer learning models with $Domain_{A}$ (Table S6. entry2) and $Domain_{AB}$ (Table S6. entry1) based on VAE FPC molecule generation model, the noveler molecules that satisfied the desired properties were generated.

Training PTL with $Domain_{A}$ was carried out on the basis of the transfer model trained with $Domain_{C}$. Based on the transfer learning model with $Domain_{D}$, model training was carried out with $Domain_{B}$, the model sampling results were shown in Table S7. Comparison with Table S6, entry2 and entry3, the number of molecules generated to meet the desired properties were increased. The reason was that the transfer model learned the pharmacologically activity in $Domain_{C}$ and $Domain_{D}$, the index that

increased more were $Lead_{request}$, which improved by 14.41% and 15.85%, respectively. However, there is a problem of low novelty.

In order to solve the above problem, this study proposed PRTL method, the sampling results was shown in Table S8, compared with Table S7, all indicators have improved, among them, the index that increased most was Novelty, which improved by 37.29% and 46.12%, respectively.

The comparative experimental results by using PRTL with AD target domain. Comparative results of that knowledge transfer from VAE_FPC molecule generation model to whole known actives and partition known actives as shown in Table S19, compare entry1 with entry2, experimental results show that in addition to Validity and Uniqueness, other indicators were improved, among them $QED_{request}$ and $Lead_{request}$ increased by 7% and 3.10% respectively.

Compare the comparative sampling result of $Domain_{AB}$ (Table S20. entry1) with the target domain was $Domain_{CD\_AB}$ (Table S19. entry2), the most improved indicator was $Lead_{request}$, it improved by 16.7%. Compared the sampling results of $Domain_{AB}$ (Table S20. entry1) with $Domain_{ABCD}$ (Table S19. entry1), QEDrequest increased by 6.03% and Novelty improved by11.41%. This was because the more focused the desired properties of molecules in the target domain are, the better the transfer learning effect is even though there are fewer molecules in the target domain under certain conditions. Compared the sampling results $Domain_{A}$ (Table S20.entry2) and $Domain_{AB}$ (Table S20 entry1), the $Lead_{request}$ was improved by 2.55%. Compare the sampling results of target domain was DomainA(Table S20, entry2) and DomainB(Table S20, entry1),

it indicate that a small number of molecules in the target domain will decrease the generation efficiency. Therefore, the target domain should satisfy the conditions that the desired properties should be focused and the number should not be too small.

Training PTL with $Domain_A$ was carried out on the basis of the transfer model trained with $Domain_C$. Based on the transfer learning model of $Domain_D$, model training was carried out with $Domain_B$, the model sampling results were shown in Table S21. Comparison with Table S20, entry2 and entry3, the number of molecules generated to meet the desired properties were increased. The reason was that the transfer model learned the biologically activity of molecules in $Domain_C$ and $Domain_D$, the index that increased most was $Lead_{request}$, an increase of 7.23% and 6.35%, respectively.

The sampling results of PRTL was shown in Table S22, compared with Table S21, all indicators have improved except for Uniqueness, among them, the index that increased most was Novelty, which further verified the effectiveness of PRTL.

Table S1. Comparison performance of different molecule generation models for CRC

	Validity	Uniqueness	$QED_{request}$	$Num_{QEDrequest}$
VAE[1]	99.38%	100.00%	51.42%	32
VAE_0.6[1]	99.53%	100.00%	92.45%	58
RNN_0.6[2]	86.88%	100.00%	87.98%	48
LSTM_0.6[3]	97.19%	100.00%	92.93%	57
GRU_0.6	97.34%	100.00%	90.02%	56
**VAE_FPC**	**100.00%**	**99.84%**	**95.61%**	**61**
CVAE[4]	99.84%	99.69%	92.29%	58

Table S2. Different molecules fingerprints based on SVM modeling method for CRCACM

Fingerprint_form	Train_Acc	Test_Acc	Train_F1	Test_F1
Rdkit_SVM	96.98%	82.09%	0.9718	0.8349
ECFP4_SVM	95.70%	80.45%	0.9601	0.8295
**Avalon_SVM**	**97.31%**	**83.42%**	**0.9751**	**0.8467**

Table S3. Different molecules fingerprints based on RF modeling method for CRCACM

Fingerprint_form	Train_Acc	Test_Acc	Train_F1	Test_F1

Rdkit_RF	98.06%	83.38%	0.9821	0.8449
ECFP4_RF	94.14%	83.89%	0.9438	0.8418
**Avalon_RF**	**97.61%**	**84.75%**	**0.9778**	**0.8565**

Table S4. Different molecules fingerprints based on GBDT modeling method for CRCACM

Fingerprint_form	Train_Acc	Test_Acc	Train_F1	Test_F1
Rdkit_GBDT	98.04%	83.40%	0.9819	0.846
ECFP4_GBDT	98.16%	84.77%	0.9830	0.8579
**Avalon_GBDT**	**98.26%**	**84.79%**	**0.9839**	**0.8584**

Table S5. Comparison performance of whole and PTL with CRC target domain

	Validity	Uniqueness	$QED_{request}$	$Lead_{request}$	Num_{Lead}	Novelty	Num_{Novel_Lead}
$Domain_{ABCD}$[3]	97.97%	94.84%	85.32%	62.62%	31	43.1%	13
$Domain_{CD\_AB}$	95.62%	96.41%	90.59%	64.41%	34	42.34%	14

Table S6. Comparison performance of PTL with CRC target DomainAB, DomainA, DomainB

	Validity	Uniqueness	$QED_{request}$	$Lead_{request}$	Num_{Lead}	Novelty	Num_{Novel_Lead}
$Domain_{AB}$	94.69%	97.03%	92.85%	61.57%	33	41.4%	13
$Domain_{A}$	93.28%	99.22%	83.17%	47.95%	23	70.97%	16
$Domain_{B}$	91.09%	98.75%	85.87%	41.85%	20	58.08%	12

Table S7. Comparison performance of PTL for CRC high and low partition activity target domain

	Validity	Uniqueness	$QED_{request}$	$Lead_{request}$	Num_{Lead}	Novelty	Num_{Novel_Lead}
$Domain_{C\_A}$	94.22%	96.09%	95.85%	62.36%	32	55.57%	17
$Domain_{D\_B}$	96.25%	96.25%	91.03%	57.70%	31	43.67%	13

Table S8. Comparison performance of PRTL for CRC high and low partition activity target domain

	Validity	Uniqueness	$QED_{request}$	$Lead_{request}$	Num_{Lead}	Novelty	Num_{Novel_Lead}
Re_A	99.53%	98.12%	99.04%	93.36%	57	92.86%	53
Re_B	99.38%	97.97%	98.88%	92.21%	56	89.79%	51

Table S9. Different molecules fingerprints based on SVM modeling method for CRCAPM

Fingerprint_ form	Train_MRE	Test_MRE	Train_MAE	Test_MAE	Train_RMSE	Test_RMSE
Rdkit_SVM	4.80%	7.86%	0.31	0.49	0.528	0.721
**ECFP4_SVM**	**3.91%**	**7.72%**	**0.25**	**0.48**	**0.426**	**0.677**
Avalon_SVM	4.23%	7.47%	0.27	0.47	0.456	0.67

Table S10. Different molecules fingerprints based on RF modeling method for CRCAPM

Fingerprint_ form	Train_MRE	Test_MRE	Train_MAE	Test_MAE	Train_RMSE	Test_RMSE
Rdkit_RF	3.73%	7.14%	0.23	0.44	0.346	0.61
ECFP4_RF	5.11%	7.13%	0.31	0.44	0.418	0.598

**Avalon_RF**	**3.39%**	**6.82%**	**0.21**	**0.42**	**0.316**	**0.598**

Table S11. Different molecules fingerprints based on GBDT modeling method for CRCAPM

Fingerprint_ form	Train_MRE	Test_MRE	Train_MAE	Test_MAE	Train_RMSE	Test_RMSE
Rdkit_GBDT	1.30%	7.06%	0.08	0.44	0.237	0.638
ECFP4_GBDT	2.38%	6.72%	0.15	0.42	0.258	0.605
**Avalon_GBDT**	**1.30%**	**6.86%**	**0.08**	**0.42**	**0.222**	**0.644**

Table S12. Comparison performance of different molecule generation models for AD

	Validity	Uniqueness	$QED_{request}$	$Num_{QEDrequest}$
VAE[1]	98.44%	99.06%	45.81%	28
VAE_0.6[1]	97.97%	100.00%	91.72%	57
RNN_0.6[2]	87.81%	100.00%	87.38%	49
LSTM_0.6[3]	95.56%	100.00%	90.14%	55
GRU_0.6	95.78%	99.83%	89.10%	54
**VAE_FPC**	**99.84%**	**99.69%**	**95.29%**	**60**
CVAE[4]	99.22%	100.00%	90.87%	57

Table S13. Different molecules fingerprints based on SVM modeling method for ADACM

Fingerprint_form	Train_Acc	Test_Acc	Train_F1	Test_F1
**Rdkit_SVM**	**99.79%**	**96.85%**	**0.9979**	**0.968**
ECFP4_SVM	100%	95.59%	1	0.954
Avalon_SVM	97.95%	95.16%	0.978	0.948

Table S14. Different molecules fingerprints based on RF modeling method for ADACM

Fingerprint_form	Train_Acc	Test_Acc	Train_F1	Test_F1
Rdkit_RF	99.95%	96.02%	0.9995	0.959
ECFP4_RF	99.69%	95.17%	0.997	0.950
**Avalon_RF**	**100.00%**	**96.85%**	**1**	**0.969**

Table S15. Different molecules fingerprints based on GBDT modeling method for ADACM

Fingerprint_form	Train_Acc	Test_Acc	Train_F1	Test_F1
Rdkit_GBDT	100%	96.01%	1	0.960
ECFP4_GBDT	100%	94.96%	1	0.948
**Avalon_GBDT**	**100%**	**96.43%**	**1**	**0.964**

Table S16. Different feature selection methods based on Rdkit_SVM for ADACM

Feature selection	Train_Acc	Test_Acc	Train_F1	Test_F1
MI	99.95%	96.85%	0.999	0.968
**Lasso**	**99.90%**	**98.32%**	**0.999**	**0.983**

Table S17. Different feature selection methods based on Avalon_RF for ADACM

Fingerprint_form	Train_Acc	Test_Acc	Train_F1	Test_F1
MI	99.90%	97.27%	0.999	0.973
**Lasso**	**100.00%**	**97.90%**	**1**	**0.980**

Table S18.Different feature selection methods based on Avalon_GBDT for ADACM

Fingerprint_form	Train_Acc	Test_Acc	Train_F1	Test_F1
MI	100.00%	96.85%	1	0.969
**Lasso**	**100.00%**	**97.27%**	**1**	**0.973**

Table S19. Comparison performance of whole and partition transfer learning with AD target domain

	Validity	Uniqueness	$QED_{request}$	$Lead_{request}$	Num_{Lead}	Novelty	Num_{Novel_Lead}
$Domain_{ABCD}$[3]	90.47%	92.81%	78.12%	70.44%	29	55.03%	16
$Domain_{CD\_AB}$	89.06%	91.56%	85.12%	73.54%	32	55.59%	18

Table S20. Comparison performance PTL with AD target DomainAB, DomainA,DomainB

	Validity	Uniqueness	$QED_{request}$	$Lead_{request}$	Num_{Lead}	Novelty	Num_{Novel_Lead}
$Domain_{AB}$	92.34%	94.84%	84.15%	56.84%	26	66.44%	17
$Domain_{A}$	92.66%	95.16%	89.7%	59.39%	30	65.25%	19
$Domain_{B}$	94.06%	94.53%	83.96%	57.91%	27	60.9%	16

Table S21. Comparison performance of PTL for AD high and low partition activity target domain

	Validity	Uniqueness	$QED_{request}$	$Lead_{request}$	Num_{Lead}	Novelty	Num_{Novel_Lead}
$Domain_{C\_A}$	90.47%	96.09%	87.35%	66.62%	32	65.22%	21
$Domain_{D\_B}$	92.5%	90.16%	82%	64.26%	28	66.43%	18

Table S22. Comparison performance of PRTL for AD high and low partition activity target domain

	Validity	Uniqueness	$QED_{request}$	$Lead_{request}$	Num_{Lead}	Novelty	Num_{Novel_Lead}
Re_A	99.69%	94.22%	99.66%	99.34%	59	90.59%	53
Re_B	98.59%	81.09%	99.62%	99.19%	50	86.98%	43

Table S23. Top20 generated molecules and the corresponding properties for CRC

Mol_name	SMILES	Pre_IC_{50}	SA
1901	CCOc1cc(-c2c[nH]nc2-c2ccc(C)cc2)cc(OC)c1OC	0.25	2.38
778	CCOc1cccc(-c2c[nH]nc2-c2cc(OC)c(OC)c(OC)c2)c1	0.37	2.42
3116	CCOc1cc(-c2c[nH]nc2-c2cc(OC)c(OC)c(OC)c2)ccc1C	0.54	2.48
3256	CC(=O)Nc1ccc(N2C(=O)NC3(CSC4=C3C(=O)c3ncccc3C4=O)C2=O)cc1	0.83	3.84
2235	CCOc1ccc(N2C(=O)NC3(CSC4=C3C(=O)c3ncccc3C4=O)C2=O)cc1	1.79	3.83
238	O=C(O)Cc1ccc(N2C(=O)NC3(CSC4=C3C(=O)c3ncccc3C4=O)C2=O)cc1	1.87	3.89
223	N#Cc1cccc(N2C(=O)NC3(CSC4=C3C(=O)c3ncccc3C4=O)C2=O)c1	1.97	4
338	O=C(O)CCc1ccc(N2C(=O)NC3(CSC4=C3C(=O)c3ncccc3C4=O)C2=O)cc1	2.01	3.87
705	CCOc1cccc(N2C(=O)NC3(CSC4=C3C(=O)c3ncccc3C4=O)C2=O)c1	2.05	3.87
666	CC(N)Cc1ccc(N2C(=O)NC3(CSC4=C3C(=O)c3ncccc3C4=O)C2=O)cc1	2.22	4.2

399	CCc1cc(C)c(N2C(=O)NC3(CSC4=C3C(=O)c3ncccc3C4=O)C2=O)cc1	2.67	3.96
397	CCOc1cc(-c2c[nH]nc2-c2cc(OC)c(OC)c(OC)c2)ccc1C(N)=O	2.86	2.58
514	Cc1cc(C)c(N2C(=O)NC3(CSC4=C3C(=O)c3ncccc3C4=O)C2=O)cc1	2.93	3.92
1474	OCc1ccc(N2C(=O)NC3(CSC4=C3C(=O)c3ncccc3C4=O)C2=O)cc1	3.26	3.89
2238	CCOc1ccc(-n2nnnc2-c2cc(OC)c(OC)c(OC)c2)cc1Cl	3.5	2.25
308	COCc1cc2c(c(OC)c1OC)-c1ccc(OC)c(=O)cc1[C@@H](NC(=O)CF)CC2	3.54	3.26
786	COCc1cc2c(c(OC)c1OC)-c1ccc(OC)c(=O)cc1[C@@H](NC(=O)CCl)CC2	3.84	3.19
1671	COc1cc(N2C(=O)NC3(CSC4=C3C(=O)c3ncccc3C4=O)C2=O)cc(OC)c1OC	4.01	3.96
2266	COc1cc(C(=O)n2nc(Nc3ccc(Cl)cc3)nc2N)cc(OC)c1OC	4.87	2.31
2140	Cc1cc2c(c(OC)c1OC)-c1ccc(OC)c(=O)cc1[C@@H](NC(=O)CF)CC2	4.97	3.15

Table S24. Top20 generated molecules and the corresponding properties for AD

Mol_name	SMILES	Dock_score	SA
652	CCc1cc(CC2COc3cc(O)ccc3C2)ccc1O	-7.492	2.84
87	C=CC1=CC(=O)CC(C)(C)C1(O)CC=Cc1ccc(O)cc1	-7.384	3.71
0	COc1ccc(C2OCC(COCc3cc(O)cc(O)c3)O2)cc1O	-7.267	3.29
426	Oc1cc(O)cc(Cc2ccc(O)cc2O)c1	-7.237	2.26
552	COC1=CC(=O)CC(C)(C)C1(O)CC=Cc1ccc(O)cc1	-7.114	3.54
548	CCc1cc(O)cc(CC)c1CCc1ccc(O)cc1	-7.049	2.2
669	CC1=CC(=O)CC(C)(C)C1(O)CC=Cc1ccc(O)cc1	-6.976	3.46
58	C=C1C[C@@H](O)CC(C)(C)C1(O)CC=Cc1ccc(O)cc1	-6.902	3.88
629	COc1cc(CC2COc3cc(O)ccc3C2)ccc1O	-6.898	2.73
386	O=c1cc(O)cc(Cc2ccc(O)cc2)o1	-6.835	2.28
5	CCc1cc(O)cc(O)c1CC	-6.817	2.34
615	COc1cc(O)cc(C[C@H]2C(=O)OC[C@@H]2Cc2ccc(O)cc2)c1	-6.786	3.18
424	CCCC1CC(=O)CC(C)(C)C1(O)CC=Cc1ccc(O)cc1	-6.782	3.77
343	COCCC1CC(=O)CC(C)(C)C1(O)CC=Cc1ccc(O)cc1	-6.753	3.8
401	Cc1c(O)cc(O)cc1CCC(=O)c1ccc(O)cc1	-6.75	2.19
590	Cc1ccc(-c2cc(=O)c3ccc(O)cc3o2)cc1O	-6.732	2.19
132	COC1=CC(=O)C2(C=C1)CC=Cc1c(O)ccc(O)c12	-6.717	4.36
398	CCCc1ccc(O)cc1CCc1ccc(O)cc1	-6.679	1.98
316	Cc1cc(CC2COc3cc(O)ccc3C2)ccc1O	-6.673	2.8
172	O=C1C[C@@H](c2ccccc2)Oc2ccc(O)cc21	-6.662	2.47

Mol_name: the name of generated molecules; SMILES: the canonical SMILES; Pre_IC_{50}: the predict value by CRCAPM; SA: the synthetic accessibility indicator (SAscore); Dock_score: the docking score between novel molecules and iNOS(for AD).

Table S25. The optimal hyper-parameters for CRCAPM, CRCACM optimized by TPE

Model_type	Main hyper-parameter	Space	Step	Distribution	CRCAPM	CRCACM
GBDT	n_estimators	[50,100]	5	Quniform	90/65/75/85/80	70/70/60/90/65
	max_depth	[5, 20]	1	Quniform	17/18/16/15/11	8/14/10/12/17
	learning_rate	[0.05,0.15]	0.01	Uniform	0.11/0.15/0.11/0.13/0.09	0.13/0.05/0.11/0.13/0.15
	subsample	[0.7, 1.0]	0.1	Uniform	0.7/0.9/1.0/0.8/0.8	0.7/0.8/0.9/1.0/0.9

Table S26. The optimal hyper-parameters for ADACM optimized by TPE

Model_type	Main hyper-parameter	Space	Distribution	ADACM
SVM	kernel	[‘rbf’, ‘sigmoid’, ‘poly’]	categorical	‘rbf’/‘rbf’/‘rbf’/poly/‘rbf’
	shrinking	[Ture, False]	categorical	True/ False/ False/ True/ False
	C	[0.001, 1000]	Uniform	977.94/587.62/397.20/380.05/81.82
	gamma	[0.0001, 8] or 1/features (0.0075)	Uniform	0.0075 /0.0075/0.0075/0.0075/0.0075

Reference

[1] Gómez-Bombarelli R, Wei JN, Duvenaud D, Hernández-Lobato JM, Sánchez-Lengeling B, Sheberla D, Aguilera-Iparraguirre J, Hirzel TD, Adams RP, Aspuru-Guzik A. Automatic Chemical Design Using a Data-Driven Continuous Representation of Molecules. *ACS Cent Sci.* 2018.

[2] M. Olivecrona, T. Blaschke, O. Engkvist, H.M. Chen. Molecular de-novo design through deep reinforcement learning. *J Cheminform*. 2017,9(1):48.

[3] F. Grisoni, B. J. H. Huisman, A. L. Button, M. Moret, K. Atz, D. Merk, G. Schneider. Combining generative artificial intelligence and on-chip synthesis for de novo drug design. *Sci Adv.* 2021, 7, eabg3338.

[4] J. Lim, S. Ryu, J.W. Kim, W.Y. Kim. Molecular generative model based on conditional variational autoencoder for de novo molecular design. *J Cheminform*, 2018, 10(1):31.

Synthetic route of compounds

Synthesis route of (4-(3-ethoxy-4,5-dimethoxyphenyl)-3-(p-tolyl)- 1H- pyrazole (compound 1901)

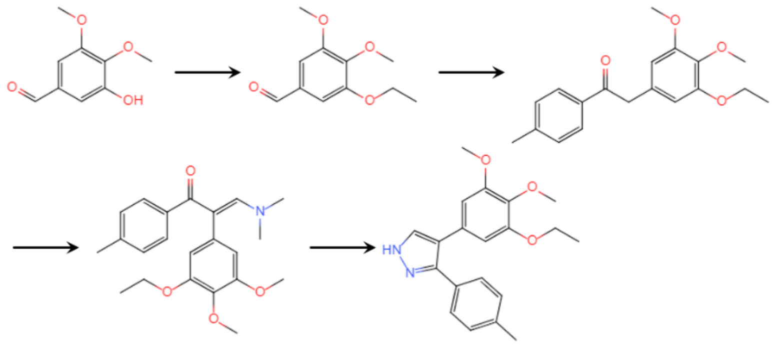

Scheme 1. Synthesis route of 1901

3-ethoxy-4,4-dimethoxybenzaldehyde

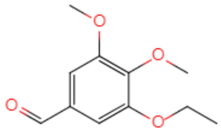

3,4-dimethoxy-5-hydroxybenzaldehyde (9.01g,49.49mmol,1eq) was added to a 250mL single-necked flask, after DMF (45ml) was dissolved, ethyl bromide (8.08g,74.15mmol,1.5eq) and cesium carbonate (24.17g,74.18mmol,1.5eq) were successively added to the reaction flask, and the reaction was carried out at room temperature. After the reaction was completed, the reaction solution was poured into 5 times the volume of water (225ml), extracted with methyl tert-butyl ether (100ml×3), dried over anhydrous sodium sulfate, and the organic solvent was removed in vacuo to obtain a yellow oily compound (88.56% yield).

2-(3-ethoxy-4,5-dimethoxyphenyl)-1-(*p*-toly)ethan-1-one

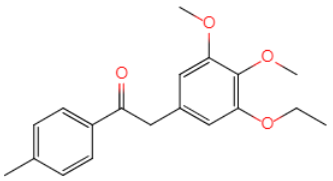

Dissolve 3-ethoxy-4,5-dimethoxybenzaldehyde (10.77g, 51.23 mmol, 1eq) in ethanol (80ml), add p-toluenesulfonylhydrazide (9.5g, 51.01mmol, 1eq), Reaction temperature at room temperature for 40 minutes. Then, an equivalent of p-methylbenzaldehyde (2.05 g, 25.80 mmol, 0.5 eq) and sodium hydroxide (3.1 g, 51.25 mmol, 1 eq) were sequentially added, and the temperature was raised to 55° C. for reaction in the dark for 24 hours. After the reaction was completed, it was cooled to room temperature, quenched by adding water, extracted with methyl tert-butyl ether 3 times (100ml×3), backwashed with water once (50ml), dried over anhydrous sodium sulfate, and the organic solvent was removed in vacuo to obtain a yellow crude oily compound, purified by column chromatography, eluent ($V_{\text{petroleum ether}}$: $V_{\text{ethyl acetate}}$ = 50:1) to obtain oily compound A2 (30.64%).

(*E*)-3-(dimethylamino)-2-(3-ethoxy-4,5-dimethoxyphenyl)-1-(*p*-tolyl)prop-2-en-1-one

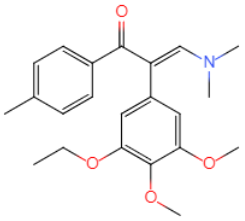

2-(3-ethoxy-4,5-dimethoxyphenyl)-1-(p-tolyl) ethan-1-one (3.1g, 9.86mmol, 1eq) was added to a 250 mL one-neck flask, and toluene was used as solvent (15ml) An equivalent of N, N-dimethylformamide dimethyl acetal (2.35 g, 19.72 mmol, 2 eq) was added, and the reaction was refluxed at 80°C under nitrogen protection. After the

reaction was completed, the solvent was removed in vacuo, a small amount of methyl tert-butyl ether was added to make a slurry, and suction filtration was performed to obtain a yellow solid compound A3 (93.94% yield).

4-(3-ethoxy-4,5-dimethoxyphenyl)-3(*p*-tolyl)-1*H*-pyrazole

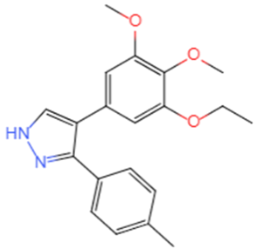

(E)-3-(dimethylamino)-2-(3-ethoxy-4,5-dimethoxyphenyl)-1-(p-tolyl)prop-2-en-1-one (3.8g, 9.92mmol, 1eq) was added to a 250mL single-necked flask, dissolved in methanol (15ml), and an aqueous solution of hydrazine hydrate (0.95, 29.64mmol, 3eq) was added, and the reaction was carried out at room temperature overnight. After the reaction was completed, methanol was removed in vacuo, a small amount of water was added to make a slurry (5ml), methyl tert-butyl ether was slurried twice (5mlX2), and suction filtration to obtain a yellow powder compound A4 (58.15% yield). ^{1}H NMR (400 MHz), Chloroform-d) δ 11.43 (s, 1H), 7.66 (s, 1H), 7.37 (d, J = 8.0 Hz, 2H), 7.16 (d, J = 7.9 Hz, 2H), 6.50 (s, 1H), 3.94 (d, J = 7.0 Hz, 2H), 3.87 (s, 3H), 3.71 (s, 3H), 3.22 (s, 1H), 2.36 (s, 3H), 1.35 (t, J = 7.0 Hz, 3H).

Synthesis route of 1-(3-chloro-4-ethoxyphenyl)-5-(3,4,5-trimethoxyphenyl) -1H-tetrazole (compound 2238)

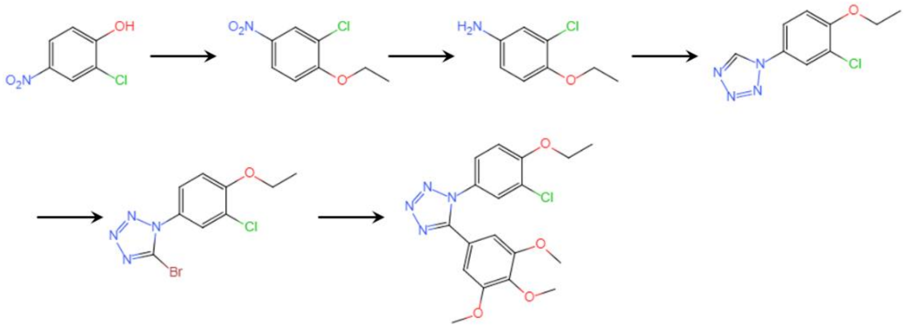

Scheme 2. Synthesis route of 2238

2-chloro-1-ethoxy-4-nitrobenzene

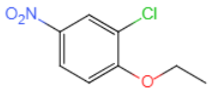

2-chloro-4-nitrophenol (20 g, 115.24 mmol, 1.0 eq.), Bromoethane (13.8 g, 126.64 mmol, 1.1 eq.), and potassium carbonate (23.89 g, 172.85 mmol, 1.5 eq.) were added in DMF for 12 h at room temperature. After the reaction, the mixture is poured into water to precipitate a large amount of yellow solid, and the mixture is filtered. The obtained filter cake is beaten with petroleum ether and purified to obtain a light yellow solid (74.01% yield).

3-chloro-4-ethoxyaniline

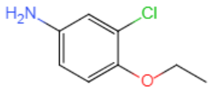

To a solution of 2-chloro-1-ethoxy-4-nitrobenzene (21g, 104.16 mmol, 1 eq.) in ethanol was added NH_4Cl (13.1g, 312.2 mmol, 3 eq.) followed by iron powder (22.8 g, 520.68 mmol, 5 eq). The reaction mixture was stirred at room temperature for 10 minutes and then heated at 50°C for 1.5 h. After cooling to room temperature, the reaction mixture was filtered and the filter cake was washed with ethanol. Concentration of the filtrate in vacuo, the concentrated filtrate was extracted with ethyl acetate and the organic layer was then dried ($MgSO_4$), and concentrated in vacuo to give the product as a white solid (80.33% yield). 1H NMR (400 MHz, CDCl3), δ: 6.85 – 6.69 (m, 1H), 6.53 (dd, J = 8.6, 2.8 Hz, 1H), 4.01 (q, J = 7.0 Hz, 1H), 3.46 (s, 1H), 1.41 (t, J = 7.0 Hz, 1H).

1-(3-chloro-4-ethoxyphenyl)-1H-tetrazole

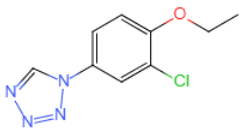

A stirred suspension of the appropriate 3-chloro-4-ethoxyaniline (12 g, 69.92 mmol, 1 eq), triethyl orthoformate (18.5 mL, 111.33 mmol, 1.6 eq.) and sodium azide (6.72 g, 103.36 mmol, 1.5 eq.) in acetic acid was refluxed for 2 h. After the reaction, the mixture was poured into water, and a large number of white solids were separated out and filtered. The filter cake obtained was washed with $NaHCO_3$ aqueous solution, and white solid compounds were obtained (79.46% yield). 1H NMR (400 MHz, CDCl3), δ: 8.92 (s, 1H), 7.75 (d, J = 2.7 Hz, 1H), 7.57 (dd, J = 8.8, 2.7 Hz, 1H), 7.10 (d, J = 8.8 Hz, 1H), 4.21 (t, J = 7.0 Hz, 2H), 1.54 (d, J = 7.0 Hz, 3H).

5-bromo-1-(3-chloro-4-ethoxyphenyl)-1H-tetrazole

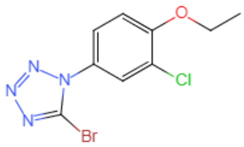

To a solution of 1-(3-chloro-4-ethoxyphenyl)-1H-tetrazole (5 g, 22.25 mmol, 1 eq.) in glacial acetic acid was added NBS (4.75 g, 26.68 mmol, 3 eq.) was refluxed for 4 h. After the reaction, the mixture was poured into water, and a large number of gray solids were separated out and filtered. The filter cake was washed with sodium bicarbonate solution and beaten twice with petroleum ether to obtain gray solid product (80.3% yield).

1-(3-chloro-4-ethoxyphenyl)-5-(3,4,5-trimethoxyphenyl)-1H-tetrazole

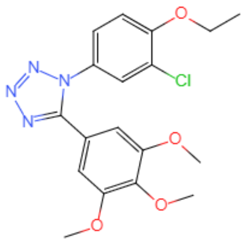

Compound 5-bromo-1-(3-chloro-4-ethoxyphenyl)-1H-tetrazole (0.5 g, 1.64 mmol, 1 eq.) was added into 250 mL trimouth and dissolved in a mixed solvent ($V_{1,\ 4\text{-dioxane}}$:V_{water} =10:1). Cesium carbonate (1.61 g, 4.94 mmol, 3 eq.) and 3,4, 5-trimethoxy phenylboric acid (0.34 g, 1.60 mmol, 1 eq.) were added, followed by palladium catalyst (0.06 g, 0.08 mmol, 0.05 eq.), and the mixture was reflow under nitrogen protection. The reaction mixture was filtered and the filter cake was washed with1, 4-dioxane. The filtrate was extracted with ethyl acetate and the organic layer was then dried ($MgSO_4$). Purify the residue by column chromatography on silica gel using EtOAc/petroleum ether as an eluent to obtain 1-(3-chloro-4-ethoxyphenyl)-5-(3,4,5-trimethoxyphenyl)-1H -tetrazole (50.2% yield). ^{1}H NMR (400 MHz, CDC13), δ: 7.54 (d, J = 2.6 Hz, 1H), 7.28 (d, J = 2.6 Hz, 1H), 7.03 (d, J = 8.8 Hz, 1H), 6.82 (s, 2H), 4.19 (q, J = 7.0 Hz, 2H), 3.89 (s, 3H), 3.73 (s, 6H), 1.53 (t, J = 7.0 Hz, 3H).

Synthesis route of 3,5-diethyl-4-(4-hydroxy phenethyl) phenol (compound 548)

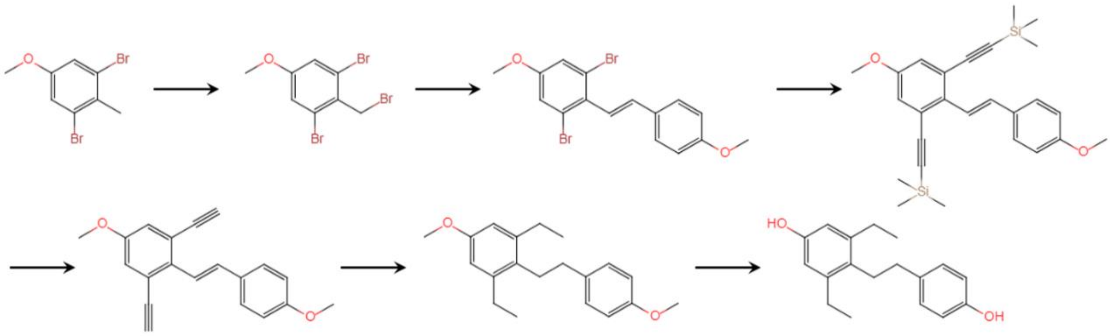

Scheme 3. Synthesis route of 548

2,6-Dibromo-4-methoxybenzyl bromide

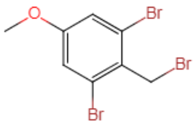

A solution of 3,5-Dibromo-4-methylanisole (5 g, 17.86 mmol, 1 eq) and NBS (3.81 g, 21.43 mmol, 1.2 eq) in 50 mL dichloroethane, AIBN (0.59 g, 3.57 mmol, 0.2 eq) was added and the mixture was stirred at 80°C for 8 hours until TLC monitoring showed completion of reactions. The solvent was quenched and evaporated under reduced pressure, the residue was dissolved in EA and washed with saturated $NaHCO_3$ (2×30 mL) solution and brine (2×20 mL). The organic phase was dried over $MgSO_4$ and concentrated in vacuum. The desired intermediate 2,6-Dibromo-4-methoxybenzyl bromide (clear oil, 90% yield) were purified by flash column chromatography (silica gel, hexane).

(E)-1,3-dibromo-5-methoxy-2-(4-methoxystyryl)benzene

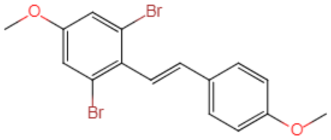

A solution of 2,6-Dibromo-4-methoxybenzyl bromide (3 g, 8.43 mmol, 1 eq) and triphenylphosphine (4.42 g, 16.86 mmol, 2 eq) in 30 mL acetonitrile was refluxed for 8 hours, then the solvent was evaporated under reduced pressure, the residue was dispersed in hexane and stirred for 2 h. The intermediate was filtered in vacuum and dried, using for next steps without any purification. Then the intermediate was

dispersed in IPA together with LiOH (0.4 g, 16.86 mmol, 2 eq), and the mixture was stirred at 80°C for 12 hours until TLC monitoring showed completion of reactions. The solvent was evaporated under reduced pressure, the residue was dissolved in EA and washed with brine (2×20 mL). The organic phase was dried over $MgSO_4$ and concentrated in vacuum. The desired intermediate (E)-1,3-dibromo-5-methoxy-2-(4-methoxystyryl)benzene (white soild, 67% yield for two steps) were purified by flash column chromatography (silica gel, hexane : EA = 8:1).

(E)-((5-methoxy-2-(4-methoxystyryl)-1,3-phenylene)bis(ethyne-2,1-diyl))bis(trimethylsilane)

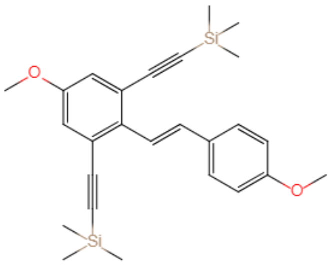

A solution of (E)-1,3-dibromo-5-methoxy-2-(4-methoxystyryl)benzene (2 g, 5.05 mmol, 1 eq), trimethylsilylacetylene (0.6 g, 6.06 mmol, 1.2 eq), $Pd(PPh_3)_2Cl_2$(0.36 g, 0.51 mmol, 0.1 eq), CuI(0.29 g, 1.52 mmol, 0.3 eq) and triethylamine(1.02 g, 10.1 mmol, 2 eq) was dissolved in dry DMF in Ar atmosphere, the mixture was stirred at 80°C for 24 hours until TLC monitoring showed completion of reactions. Then diluted with water and extracted with EA. The organic phase was dried over $MgSO_4$ and concentrated in vacuum. The desired intermediate (E)-((5-methoxy-2-(4-methoxystyryl)-1,3-phenylene)bis (ethyne-2,1-diyl))bis(trimethylsilane) (yellow oil, 62% yield) were purified by flash column chromatography (silica gel, hexane : EA = 5:1).

(E)-1,3-diethynyl-5-methoxy-2-(4-methoxystyryl)benzene

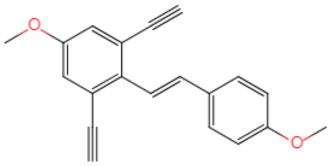

A solution of (E)-((5-methoxy-2-(4-methoxystyryl)-1,3-phenylene)bis(ethyne- 2,1-diyl))bis(trimethylsilane) (1.5 g, 3.47 mmol, 1 eq) in THF was added TBAF(1.09 g, 4.16 mmol, 1.2eq) dropwise, the mixture was stirred at room temperature for 4 hours. The reaction was quenched with water and diluted with EA. The organic phase was collected and dried over $MgSO_4$ and concentrated in vacuum. The desired intermediate (E)-1,3-diethynyl-5-methoxy-2-(4-methoxystyryl)benzene (yellow oil, 83% yield) were purified by flash column chromatography (silica gel, hexane : EA = 6:1).

1,3-diethyl-5-methoxy-2-(4-methoxyphenethyl)benzene

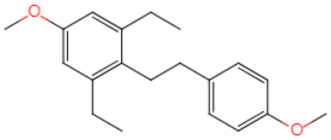

A solution of (E)-1,3-diethynyl-5-methoxy-2-(4-methoxystyryl)benzene (1g, 3.47 mmol, 1 eq) in methanol, 10% Pd/C (0.4g) was added and the mixture was reacted at room temperature in hydrogen atmosphere until TLC monitoring showed completion of reactions. The filtrate was collected and concentrated in vacuum. The desired intermediate 1,3-diethyl-5-methoxy-2- (4-methoxyphenethyl)benzene (white solid, 90% yield) were purified by flash column chromatography (silica gel, hexane : EA = 8:1).

3,5-diethyl-4-(4-hydroxyphenethyl)phenol

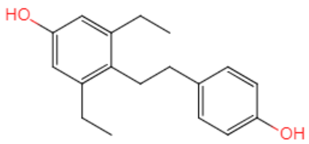

A solution of 1,3-diethyl-5-methoxy-2-(4-methoxyphenethyl)benzene (1g, 3.35 mmol, 1 eq) in dry DCM, BBr_3 (1.68 g, 6.7 mmol, 2 eq) was added dropwise at 0°C in Ar atmosphere, the mixture was stirred at room temperature for 4 hours until TLC monitoring showed completion of reactions. The reaction was quenched with water and extracted with EA. The organic phase was collected and dried over $MgSO_4$ and concentrated in vacuum. The desired 3,5-diethyl-4-(4-hydroxyphenethyl)phenol (pale white solid, 92% yield) were purified by flash column chromatography (silica gel, hexane : EA = 4:1).

Synthesis route of 3-(4-hydroxyphenethyl)-4-propylphenol (compound 398)

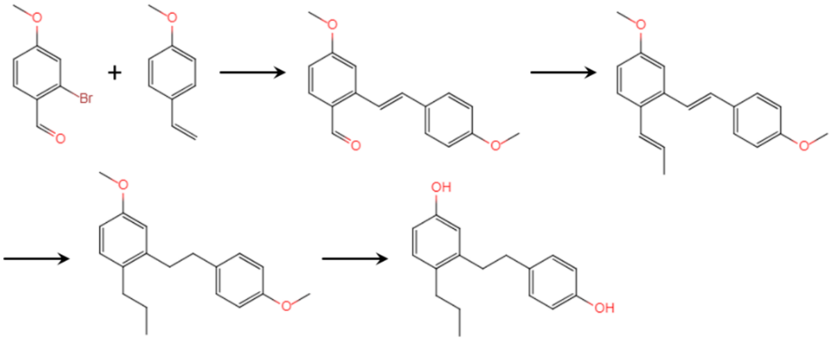

Scheme 4. Synthesis route of 398

(E)-4-methoxy-2-(4-methoxystyryl)benzaldehyde

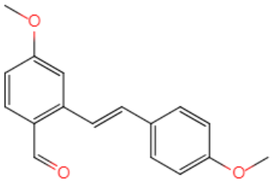

A solution of 2-bromo-4-methoxybenzaldehyde(3g, 14.02 mmol, 1 eq), 4-

methoxystyrene(2.26 g, 16.82 mmol, 1.2 eq), Tris(dibenzylideneacetone) dipalladium (2.56 g, 2.80 mmol, 0.2 eq), *t*-BuOK(3.15 g, 28.04 mmol, 2 eq) in dry DMF. The mixture stirred at 80°C in Ar atmosphere for 20 hours until TLC monitoring showed completion of reactions. The reaction was diluted with water and extracted with EA. The organic phase was collected and dried over $MgSO_4$ and concentrated in vacuum. The desired intermediate (E)-4-methoxy-2-(4-methoxystyryl)benzaldehyde (white solid, 81.4% yield) were purified by flash column chromatography (silica gel, hexane : EA = 8:1).

4-methoxy-2-((E)-4-methoxystyryl)-1-((E)-prop-1-en-1-yl)benzene

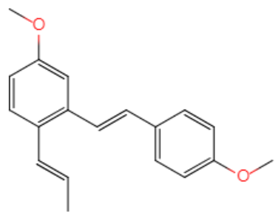

A solution of (E)-4-methoxy-2-(4-methoxystyryl)benzaldehyde (2g, 7.46 mmol, 1 eq), Ethyltriphenylphosphonium bromide (3.32 g, 8.95 mmol, 1.2 eq), *t*-BuOK(1.67 g,14.92 mmol, 2 eq) in THF. The mixture stirred at room temperature in Ar atmosphere for 6 hours until TLC monitoring showed completion of reactions. The reaction was diluted with water and extracted with EA. The organic phase was collected and dried over $MgSO_4$ and concentrated in vacuum. The desired intermediate 4-methoxy-2-((E)-4-methoxystyryl)-1- ((E)-prop-1-en-1-yl)benzene (white solid, 76.5% yield) were purified by flash column chromatography (silica gel, hexane : EA = 6:1).

4-methoxy-2-(4-methoxyphenethyl)-1-propylbenzene

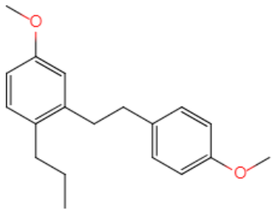

A solution of 4-methoxy-2-((E)-4-methoxystyryl)-1-((E)-prop-1-en-1-yl)benzene (1g, 3.57 mmol, 1 eq) in methanol, 10% Pd/C (0.4g) was added and the mixture was reacted at room temperature in hydrogen atmosphere until TLC monitoring showed completion of reactions. The filtrate was collected and concentrated in vacuum. The desired intermediate 4-methoxy-2-(4- methoxyphenethyl)-1-propylbenzene (white solid, 89.5% yield) were purified by flash column chromatography (silica gel, hexane : EA = 6:1).

3-(4-hydroxyphenethyl)-4-propylphenol

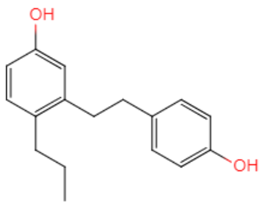

A solution of 4-methoxy-2-(4-methoxyphenethyl)-1-propylbenzene (1g, 3.52 mmol, 1 eq) in dry DCM, BBr_3 (1.76 g, 7.04 mmol, 2 eq) was added dropwise at 0°C in Ar

atmosphere, the mixture was stirred at room temperature for 2 hours until TLC monitoring showed completion of reactions. The reaction was quenched with water and extracted with EA. The organic phase was collected and dried over $MgSO_4$ and concentrated in vacuum. The desired 3-(4-hydroxyphenethyl)-4-propylphenol (pale white solid, 94.3% yield) were purified by flash column chromatography (silica gel, hexane : EA = 5:1).
