# Supplementary figures and images for: De Novo Generation and Identification of Novel Compounds with Drug Efficacy Based on Machine Learning
Source: Adv Sci (Weinh). 2024 Jan 10;11(11):2307245. doi: 10.1002/advs.202307245 (PMC10962488; doi:10.1002/advs.202307245)

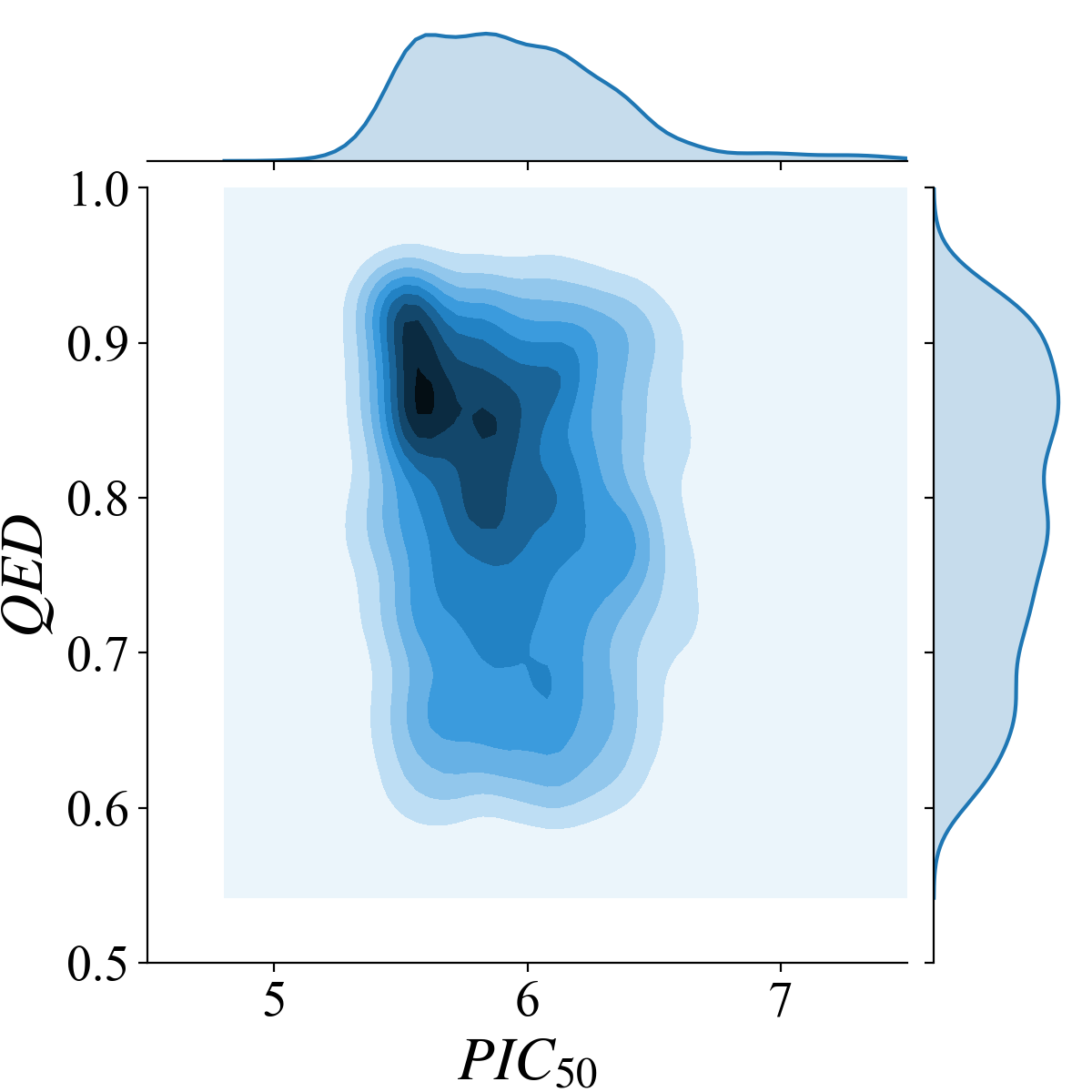

Supplement: Supplementary file 3 — Supporting Information [file ADVS-11-2307245-s001.zip › data7/Generate_Distribution_joint_QED_ic50.png]
