# Supplementary material for: De Novo Generation and Identification of Novel Compounds with Drug Efficacy Based on Machine Learning
Source: Adv Sci (Weinh). 2024 Jan 10;11(11):2307245. doi: 10.1002/advs.202307245 (PMC10962488; doi:10.1002/advs.202307245)
Supplement: Supplementary file 3 — Supporting Information [file ADVS-11-2307245-s001.zip › DTLS/README.pdf]

De Novo Generation and Identification of Novel Compounds with Drug Efficacy Based on Machine Learning

This repo contains the source code for **De Novo Generation and Identification of Novel Compounds with Drug Efficacy Based on Machine Learning**

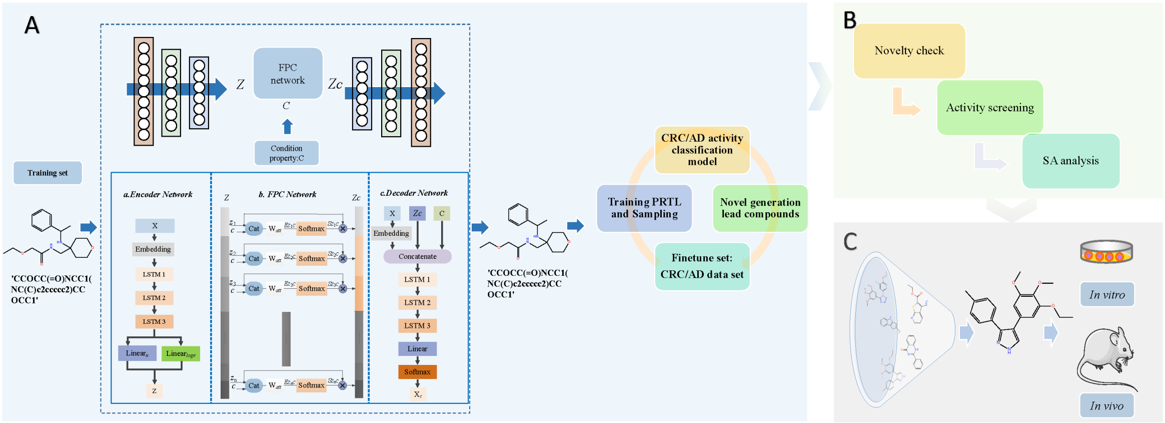

Requirements

- Python 3.6
- [rdkit 2018.03.3]
- [pytorch 1.7.1]
- Other dependencies noted in `requirements.txt`, please install them with `pip`.

The package should take approximately 120 seconds to install on a recommended computer.

The package development version is tested on Ubuntu 16.04 and Windows10.

Demo

Take CRC experiment as example （since the upload system limits the size to 40MB, the data dataset is divided into subfolders, which need to be placed under the data folder after unzipped to run， And put the 'prior encoder.ckpt' of ‘data4’ and ‘data6’ into ‘data3/two_finetune_C_A_early_results/2run_2021-09-06-23_08_25 ’ and ‘data5/two_finetune_D_B_early_results/2run_2021-09-06-22_18_24’， respectively）：

Step 1. Train the PRTL with high activity sub-partition target domain, so as to generate the novel molecules that satisfy the chemically valid, drug-like and activity against CRC. Please run:

```
python train_PRTL_A.py --test_file=data/Filer_ChEMBL_JCA_dataset.txt --prop_file=data/Recurrent_datasetA.txt --prior_model_path=data/two_finetune_C_A_early_results/2run_2021-09-06-23_08_25 --voc_file=data/train_voc.txt
```

- test_file: the combine dataset of CRC target domain and the training dataset, which used for screen molecules in every fine-tune
- prop_file: the save data file, which initial data is the target domain of the PTL. In PRTL process, the generated novel molecules are saved to achieve the update of the target

domain

- prior_model_path: pretrain model parameters, which as the initial model parameters of transfer learning
- voc_file:the alphabet of train model

Step 2. Train the PRTL with low activity sub-partition target domain, so as to generate the novel molecules that satisfy the chemically valid, drug-like and activity against CRC. The PRTL with low activity sub-partition target domain, the overall training process is similar to step 1, which only needs to change its file path of initial model parameters, initial target domain and save the model. Please run:

```
python train_PRTL_B.py --test_file=data/Filer_ChEMBL_JCA_dataset.txt --prop_file=data/Recurrent_datasetB.txt --prior_model_path=data/two_finetune_D_B_early_results/2run_2021-09-06-22_18_24 --voc_file=data/train_voc.txt
```

Step 3. Gain novel molecules were deduplicated with the known data set and saved for subsequent studies. Please run:

```
python Gain_Novel_molecules.py
```

Step 4. Calculate the physicochemical and biological properties of the generated novel molecules, e.g the predicted Pic50 and ic50 values and SAscore, QED, MW, HBA, HBD etc physicochemical properties. Please run:

```
python get_properties.py
```

Note: Due to the uncertainty of the model, the samples results may be slightly different each time, so this study submitted the running results to the system. If rerun the code, please keep the original data and files.

Results

The output is the generate novel molecules that satisfy the chemical valid, drug-like and activity against CRC, which save in `Recurrent_A_dataset.txt` and `Recurrent_B_dataset.txt`, the molecules that not in the target domain molecule and the training set was retained, the mol file of which was saved in `Generate_JCA_molecules`, and corresponding molecule image was saved in `Generate_pictures`. The joint KDE curve of QED and predicted pic50 as shown below, the properties of generate novel molecules was obtained in `Generate_properties.csv`

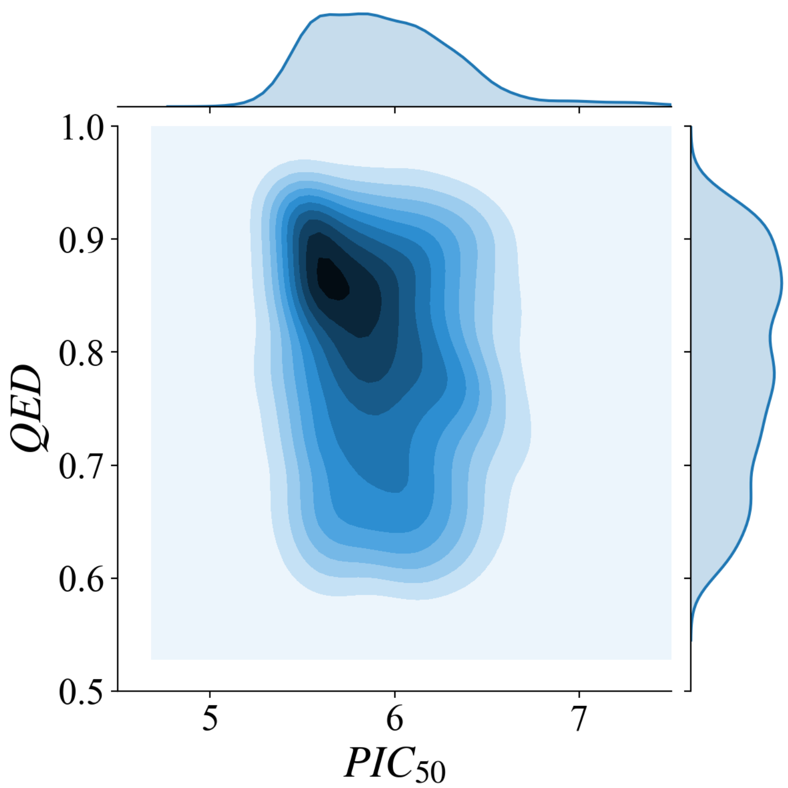
1.0
0.9
0.8
0.7
0.6
0.5
QED
5
6
7
PIC50
